# HSP90 identified by a proteomic approach as druggable target to reverse platinum resistance in ovarian cancer

**DOI:** 10.1002/1878-0261.12883

**Published:** 2021-01-19

**Authors:** Rita Lombardi, Maura Sonego, Biagio Pucci, Laura Addi, Federica Iannelli, Francesca Capone, Luigi Alfano, Maria Serena Roca, Maria Rita Milone, Tania Moccia, Alice Costa, Elena Di Gennaro, Francesca Bruzzese, Gustavo Baldassarre, Alfredo Budillon

**Affiliations:** ^1^ Experimental Pharmacology Unit‐Laboratories of Naples and Mercogliano (AV) Istituto Nazionale per lo Studio e la Cura dei Tumori “Fondazione G. Pascale” – IRCCS Naples Italy; ^2^ Division of Molecular Oncology Centro di Riferimento Oncologico di Aviano (CRO) IRCCS Aviano Italy; ^3^ Cell Biology and Biotherapy Unit Istituto Nazionale Tumori ‐ IRCCS, Fondazione G. Pascale Naples Italy; ^4^ University of Trieste Italy

**Keywords:** cisplatin, drug resistance, HSP90, ovarian cancer, proteomics

## Abstract

Acquired resistance to platinum (Pt)‐based therapies is an urgent unmet need in the management of epithelial ovarian cancer (EOC) patients. Here, we characterized by an unbiased proteomics method three isogenic EOC models of acquired Pt resistance (TOV‐112D, OVSAHO, and MDAH‐2774). Using this approach, we identified several differentially expressed proteins in Pt‐resistant (Pt‐res) compared to parental cells and the chaperone HSP90 as a central hub of these protein networks. Accordingly, up‐regulation of HSP90 was observed in all Pt‐res cells and heat‐shock protein 90 alpha isoform knockout resensitizes Pt‐res cells to cisplatin (CDDP) treatment. Moreover, pharmacological HSP90 inhibition using two different inhibitors [17‐(allylamino)‐17‐demethoxygeldanamycin (17AAG) and ganetespib] synergizes with CDDP in killing Pt‐res cells in all tested models. Mechanistically, genetic or pharmacological HSP90 inhibition plus CDDP ‐induced apoptosis and increased DNA damage, particularly in Pt‐res cells. Importantly, the antitumor activities of HSP90 inhibitors (HSP90i) were confirmed both *ex vivo* in primary cultures derived from Pt‐res EOC patients ascites and *in vivo* in a xenograft model. Collectively, our data suggest an innovative antitumor strategy, based on Pt compounds plus HSP90i, to rechallenge Pt‐res EOC patients that might warrant further clinical evaluation.

## Graphical abstract

By unbiased proteomics, we identified HSP90 as a central hub in platinum (Pt)‐resistant ovarian cancer models. Indeed, genetic and pharmacologial HSP90 inhibition sensitized resistant cells to CDDP treatment, potentiating DNA‐damage and apoptosis. Combined CDDP plus anti‐HSP90 therapy is effective also *ex vivo* on primary cultures from Pt‐resistant (Pt‐res) ovarian cancer patients and *in vivo* Pt‐res ovarian cancer xenograft mouse model.

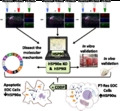

**Abbreviations**
17AAG: 17‐(Allylamino)‐17‐demethoxygeldanamycin
2‐D DIGE: two‐dimensional differential in‐gel electrophoresis
CDDP: cisplatin
CI: combination index
DIA: differential in‐gel analysis
DRI: dose reduction index
EOC: epithelial ovarian cancer
HGSOC: high‐grade serous ovarian cancers
HSP90: heat‐shock protein 90
HSP90i: HSP90 inhibitors
HSP90α: heat‐shock protein 90 alpha isoform
i.p.: intraperitoneal
IPA: ingenuity pathway analysis
LC‐MS/MS: liquid chromatography–mass spectrometry tandem
NSG: NOD scid gamma
PCA: principal component analysis
Pt: platinum
Pt‐res: Pt‐resistant
TGD: tumor growth delay
TV: tumor volume

## 1 Introduction

Epithelial ovarian cancer (EOC) represents the seventh most commonly diagnosed cancer among women and the most lethal gynecological disease, with a 5‐year relative survival rate of 46% after the diagnosis [1, 2]. EOC is not a single disease but comprises different histological entities (e.g., high‐grade serous, endometrioid, mucinous, clear cell, and low‐grade serous) with different biological, molecular, and clinical characteristics and high‐grade serous ovarian cancers (HGSOC) are the most common type of EOC, accounting for 75% of all EOC [3]. The standard treatment of advanced EOC is based on the maximum debulking surgery, followed by platinum (Pt)‐based chemotherapy, which remained the same over the past three decades. Many anticancer agents, including molecular‐targeted agents and combination therapies, have been developed and validated clinically in Pt‐sensitive EOC patients [3]. However, the overall survival rate has not been improved significantly in the metastatic disease. Indeed, although EOC is one of the most chemo‐responsive tumors, almost invariably after an initial response, the majority of patients relapse and Pt resistance invariably emerges [4, 5]. Therefore, understanding the underlying molecular mechanisms associated with the onset of Pt resistance is an urgent unmet clinical need.

On this regard, we previously generated and characterized three CDDP‐resistant isogenic high‐grade EOC cell lines (TOV‐112D Pt‐res, MDAH‐2774 Pt‐res, and OVSAHO Pt‐res) from their parental counterparts [6, 7, 8]. Since gene expression profiling failed to identify signaling pathways commonly altered in Pt‐resistant (Pt‐res) cells [6], we took advantage of a proteomic approach to investigate the possible mechanisms by which cells acquire CDDP resistance. We highlighted the heat‐shock protein 90 (HSP90) as a central hub of the network of proteins differentially expressed between resistant and parental cells in all the three ovarian cancer models explored. Small‐molecule inhibitors for the HSP90 have been extensively exploited in preclinical studies representing promising agents in cancer treatment and some of them, such as tanespimycin (17AAG) and ganetespib (STA9090), are in phase II/III clinical studies in cancers patients alone or in combination with conventional chemotherapy^2^. Therefore, we tested either 17AAG or ganetespib activity in Pt‐res ovarian cancer cells demonstrating a strong synergistic antitumor effect when used in combination with CDDP *in vitro* and *in vivo* xenograft models as well as in primary cultures from Pt‐res ovarian cancer patients.

## 2 Materials and methods

### 2.1 Cell culture and cisplatin‐resistant cell selection

TOV‐112D (CRL‐11731), MDAH‐2774 (CRL‐10303) cells and hTERT‐immortalized human foreskin fibroblasts BJ‐hTERT (CRL‐4001) were from ATCC (Manassas, VA, USA) while OVSAHO (JCRB1046) cells were from JCRB Cell Bank (Tokyo, Japan). BJ‐hTERT cells were cultured in Dulbecco's modified Eagle's medium, whereas TOV‐112D, MDAH‐2774, and OVSAHO were cultured in RPMI‐1640 medium. All media were supplemented with 10% FBS (Cambrex, Verviers, Belgium) heat‐inactivated, 50 units per mL penicillin (Cambrex), 500 g·mL^−1^ streptomycin (Cambrex), and glutamine 4 mm. The cells were grown in a humidified atmosphere composed of 95% air and 5% CO_2_ at 37 °C.

CDDP‐resistant cancer cells (referred as Pt‐res cells) were generated as previously described and include pooled resistant populations (pool) and individual clones, as indicated in the text [6, 7, 8]. Primary cells (OV102, OV202, OV199, OV200) were established from ascites of EOC patients, collected by the CRO‐Aviano National Cancer Institute Institutional Biobank with a written informed consent from patients. The CRO‐Aviano Internal Review Board approved this study (#IRB‐06/2011).

Primary cells were maintained in OCMI medium (M199 and Ham's 1 : 1) supplemented with 2% FBS, EGF (10 ng·mL^−1^), hydrocortisone (500 ng·mL^−1^), cholera toxin (25 ng·mL^−1^), and insulin (20 µm·mL^−1^). Negative mycoplasma cultures were confirmed by monthly mycoplasma tests.

### 2.2 2‐D DIGE (two‐dimensional differential in‐gel electrophoresis), image acquisition, analysis, and processing

All 2‐D DIGE reagents and instruments were provided by GE Healthcare (Pittsburgh, PA, USA). Proteomic experiments were performed as described by Milone *et al*. [9]. In detail, four independent biological conditions are required to give statistic confidence to 2‐D DIGE data. All proteomics maps were subjected to the following two analysis modules from the DeCyder™ version 7.2 software (GE Healthcare): differential in‐gel analysis (DIA) and biological variation analysis. Matched spots considered for the analysis were filtered according to the following criteria: their presence was detected in at least 80% of spot maps, the average ratio selected was ≤ 1.3 or ≥ 1.3, and unpaired Student's *t*‐test. Protein expression changes were statistically supported by a p < 0.05. In addition, an unsupervised multivariate analysis was carried out by the extended data analysis module, and based on differential protein expression profiles, the overall correlations occurring between the spot maps were visualized using a principal component analysis (PCA).

### 2.3 Protein identification by liquid chromatography–mass spectrometry tandem (LC‐MS/MS)

Protein extracts were separated on preparative gels and recovered from the gels for identification by LC‐MS/MS as previously described [9]. Mass fingerprinting searching was carried out in Swiss‐Prot/TrEMBL database using Mascot (Matrix Science Ltd., London, UK).

### 2.4 Immunoblotting

Protein extraction after 48 h of cell culture and immunoblotting was performed as previously described [9]. Western blots were quantified using imagej software (Rasband, W.S., U.S., National Institutes of Health, Bethesda, Maryland, USA). Primary antibodies were purchased as follows: HSP90 alpha 2G5.G3 (#SMC‐147) and HSP90 (total) 4F3.E8 (#SMC‐149) from StressMarq Biosciences (Victoria, BC, Canada); GRP75 (#3593), LMN A/C (#2032), ANXA1 (#3299), Bax (#2774), CASPASE 3 (#9662), cleaved PARP1 (Asp214; #5625), PARP1 (#9542), Bcl‐2 (#4223S) from Cell Signaling Technology (Leiden, Netherlands); VIM (ab 16700), prohibitin (PHB; ab55618), CALR (ab2907), heat‐shock cognate 71‐kDa protein (HSP7C; ab19136), PGK1 (ab67335), PRDX4 (ab15574) from Abcam (Cambridge, UK); GRP78 C‐20 (sc‐1051), EZR H‐276 (sc‐20773), β‐actin C4 (sc‐47778), and GAPDH (FL‐335) from Santa Cruz Biotechnology Inc., (Dallas, TX, USA); VINC (10C‐CR1199M1) from Fitzgerald (Acton, MA, USA); ɣH2AX (Ser139) clone JBW301 (#05‐636) from Millipore (Burlington, MA, USA); and heterogeneous nuclear ribonucleoprotein L (HNRPL; A303‐895A) from Bethyl (Montgomery, TX, USA).

Secondary antibodies were purchased as follows: polyclonal swine anti‐rabbit immunoglobulins/horseradish peroxidase (HRP)‐linked IgG secondary antibody conjugate and polyclonal rabbit anti‐goat immunoglobulins/HRP conjugate from Dako Products, Agilent (Santa Clara, CA, USA); rabbit polyclonal anti‐mouse IgG H&L (HRP) conjugate from Abcam.

### 2.5 Protein network analyses

The MS‐identified proteins, whose expression changes, were analyzed by (ingenuity pathway analysis) ipa software (GeneGo Inc., St. Joseph, MI, USA). IPA includes a manually annotated database of protein interactions and metabolic reactions obtained from the scientific literature. The networks were graphically visualized as hubs (proteins) and edges (the relationship between proteins).

### 2.6 HSP90α knockout

The TOV‐112D heat‐shock protein 90 alpha isoform (HSP90α) knockout cells were generated through the CRISPR‐Cas9 system as reported previously [10]. Briefly, cells were transfected with the pSpCas9 (BB)‐2A‐Puro (PX459) V2.0 [11] (gift from Feng Zhang, Addgene plasmid #62988) containing guide RNAs targeting the HSP90 exon three (5′‐GATCAAAAGGAGCACGTCGT‐3′) and selected with 5 g·mL^−1^ of puromycin. The generated cell clones were analyzed by western blot and sequencing to verify knockout of HSP90.

### 2.7 Cell proliferation assay and drug combination studies

*cis*‐Diammine Pt (II) dichloride (CDDP) was provided by Sigma‐Aldrich (St. Louis, MO, USA); carboplatin was provided by Teva (Milan, Italy). Stock solutions were prepared in PBS. 17AAG, a derivative of geldanamycin (GM), was provided by StressMarq Biosciences. Stock solutions were prepared in DMSO. Ganetespib was provided by MedChem Express (Sollentuna, Sweden), and stock solutions were prepared in DMSO.

Cell proliferation was measured in 96‐well plates in cells untreated and treated with 17AAG, ganetespib and CDDP as single agent or in combination. Cell proliferation was measured using a spectrophotometric dye incorporation assay (sulforhodamine) [12]. Drug combination studies were based on concentration–effect curves generated as a plot of the fraction of unaffected (surviving) cells *vs* drug concentration after 72 or 96 h of treatment. Synergism, additivity, and antagonism were quantified after an evaluation of the combination index (CI), which was calculated by the Chou–Talalay equation with calcusyn software (Biosoft, Cambridge, UK), as described elsewhere [13]. A CI ≤ 0.8, CI ≤ 0.9, CI = 0.9–1.1, and CI > 1.1 indicated a strong synergistic, a synergistic, an additive, or an antagonistic effect, respectively. The dose reduction index (DRI) determines the magnitude of dose reduction allowed for each drug when given in combination, compared with the concentration of a single agent that is needed to achieve the same effect.

For dose–response curves, primary EOC cells were seeded in 96‐well culture plates and treated with increasing doses of CDDP with or without ganetespib (25 nm) for 72 h. Cell viability was analyzed by MTS assay using the CellTiter 96 AQueous cell proliferation assay kit (Promega, Madison, WI, USA).

### 2.8 RNA isolation and Real‐time PCR

Total RNA was isolated from parental and Pt‐res cells using TriZol reagent (Ambion, Austin, TX, USA) following the manufacturer’s instructions. One micrograms of total RNA was retro‐transcribed using random hexamers and the AMV reverse transcriptase (Promega). Primers used were the following: for human HSP90α (forward 5′‐GCAGGGCAACACCTCTACAA‐3′ and reverse 5′‐GTCTTGGGTCTGGGTTTCCT‐3′), HSP‐90β (forward 5′‐cgcatgaaggagacacagaa‐3′ and reverse 5′‐tcccatcaaattccttgagc‐3′), and human GAPDH (forward 50‐GAAGGTGAAGGTCGGAGTC‐30 and reverse 50‐GAAGATG GTGATGGGATTTC‐30). Quantitative real‐time PCR analyses were performed using the CFX96 TM real‐time PCR detection system (Bio‐Rad Laboratories, Inc., Hercules, CA, USA).

### 2.9 Colony formation assay

Five hundred cells per well were plated in 6‐well plates. Cells were treated concomitantly or with 24 h of delay with different concentrations (IC_10_–IC_25_) of ganetespib and CDDP and were allowed to grow for about 10 days until colonies could be clearly seen. Cell culture plates containing colonies were gently washed with PBS and fixed and stained with crystal violet 0.5%, methanol 20% for 30 min. Excess stain was removed by washing repeatedly with distilled water. Crystal violet was eluted with 100% methanol. Absorbance was read at 595 nm. All the procedures were done at room temperature.

### 2.10 Apoptosis assays

Apoptosis and necrosis were identified by flow cytometry analysis. Briefly, 3 × 10^5^ cells were seeded and after 48 or 72 h of treatment were stained with Annexin V‐FITC and propidium iodide, following the manufacturer's instructions (MACS; MiltenyiBiotec, Bergisch Gladbach, Germany) and analyzed by flow cytometry.

### 2.11 *In vivo* xenograft studies

All studies have been performed in compliance with institutional guidelines and regulations (Directive 2010/63/EU; Italian Legislative Decree DLGS 26/2014). Female NOD scid gamma (NSG) mice (4–6 weeks old) and female athymic (nude) mice (4–6 weeks old) were acquired from Charles River Laboratories (Charles River, Wilmington, MA, USA) and used for TOV‐112D Pt‐res pool 2 and TOV‐112D parental xenograft model, respectively. Mice were acclimatized in the Animal Care Facility of ‘Fondazione G. Pascale’‐IRCCS/Laboratori di Mercogliano‐CROM. After 3 days, TOV‐112D Pt‐res pool 2 cells (5 × 10^6^) diluted in 200 mL [PBS/Matrigel GF (Becton Dickinson, Franklin Lakes, NJ, USA) 1/1] were injected subcutaneously (s.c) in flank regions of the mice. When the tumors became palpable, the mice were randomized into four experimental groups (*n* = 5). Intraperitoneal (i.p.) treatment was performed with CDDP (2.5 mg·kg^−1^ dissolved in PBS) and/or ganetespib (75 mg·kg^−1^ dissolved in 10% DMSO + 40% poly(ethylene glycol) + 50% ddH_2_O), or vehicles, once a week. TOV‐112D parental cells (3 × 10^6^) were injected s.c in both flank regions of the nude mice. When the tumors became palpable, the mice were randomized into four experimental groups (*n* = 4). I.p. treatments were performed with carboplatin (CBDCA; 15 mg·kg^−1^ dissolved in PBS) and/or ganetespib (30 mg·kg^−1^ dissolved in 10% DMSO + 40% poly(ethylene glycol) + 50% ddH_2_O), or vehicles, three times/week. Mice in the control groups were treated with both PBS and 10% DMSO + 40% poly(ethylene glycol) + 50% ddH_2_O. Tumor volume (TV; mm^3^) and tumor growth delay (TGD) were compared with that of the vehicle control group as described before [13]. Overall, we took adequate steps to insure that animals did not suffer unnecessarily at any stage of the experiment described above.

### 2.12 Statistical analysis

All experiments were performed at least three times. Statistical significance was determined by one‐way ANOVA, or by a two‐tailed unpaired Student's *t*‐test, as indicated and a *P* < 0.05 was considered to be statistically significant. All statistical evaluations were performed with graphpad prism 8 (Ritme Informatique, Paris, France).

## 3 Results

### 3.1 Proteomic analysis identified several differentially expressed proteins between Pt‐resistant and parental EOC cells

**Fig. 1:**
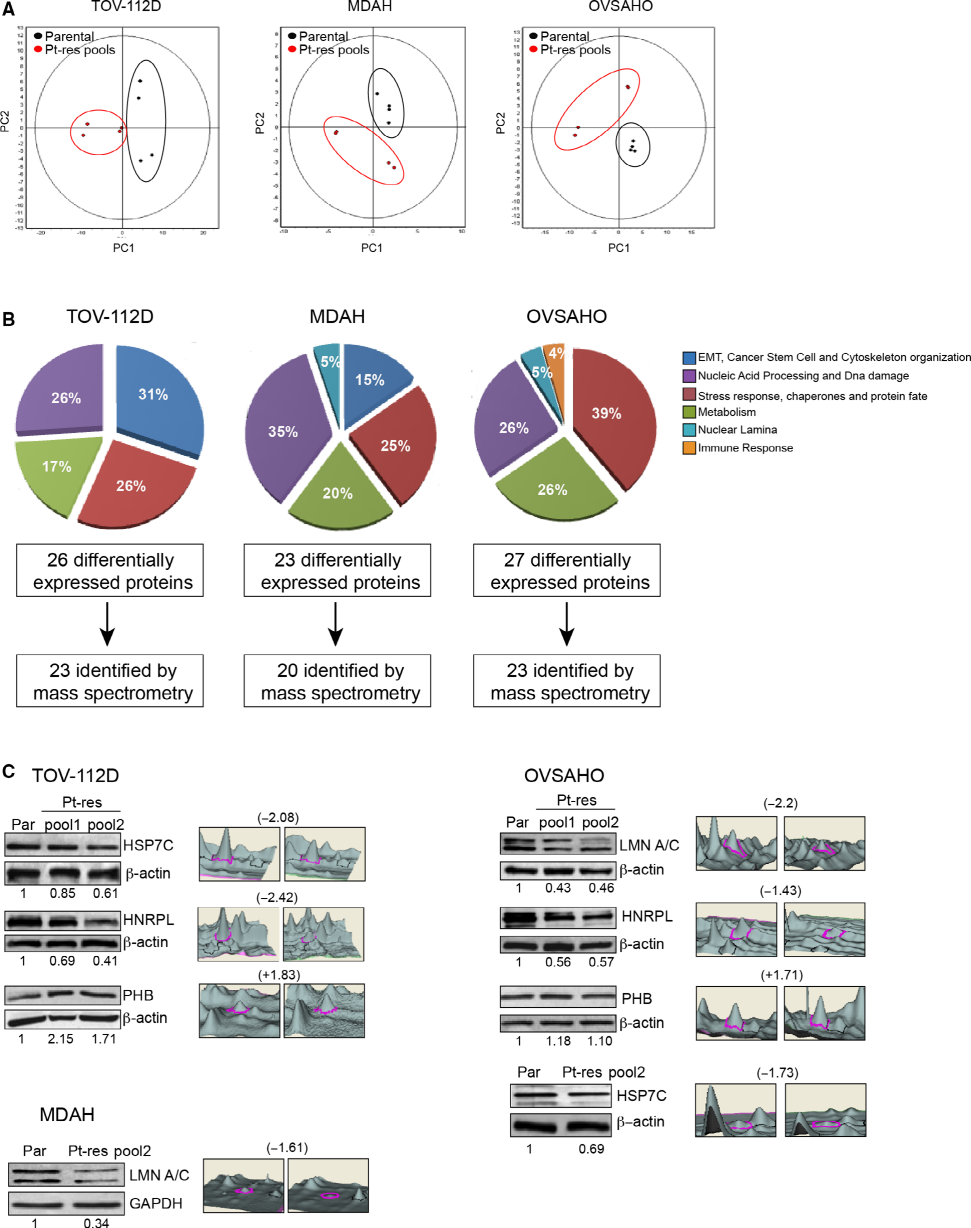
Comparative proteomic analysis of three CDDP‐resistant isogenic EOC cells (TOV‐112D Pt‐res pool, MDAH Pt‐res pool, and OVSAHO Pt‐res pool) *vs* parental cells. (A) Unsupervised multivariate analysis (PCA plots) showed good experimental reproducibility as demonstrated by the close relation between the four biological replicates of either parental cells or pooled resistant populations (two different pools for each Pt‐res cell line) in the 2‐D DIGE results. The eight spot maps for each cell line clearly clustered into two groups corresponding to Pt‐res pool cells (red) *vs* parental cells (black; *P* < 0.05). *P*‐values were calculated based on unpaired Student's *t*‐test. (B) Functional distribution of proteins identified by 2‐D DIGE LC‐MS/MS is showed by the pie charts and the number of differentially expressed proteins, as well as the number of identified proteins is reported. (C) Western blot validation of four of the eight proteins differentially expressed by at least two cell lines. The 3D view of 2‐D DIGE quantification for each spot is reported. The numbers indicate the fold change values in protein spot levels, from 2‐D DIGE data, relative to parental cells, as reported in Tables S1‐S3. β‐Actin and GAPDH were used as loading controls. Western blot quantification was performed by imagej software.

To investigate the mechanism by which cells acquire Pt resistance, we took advantage of 2‐D DIGE followed by LC‐MS/MS to compare the protein expression profile of the three CDDP‐resistant cells (TOV‐112D Pt‐res pool, OVSAHO Pt‐res pool, and MDAH‐2774 Pt‐res pool) that we have previously established and characterized [6, 7, 8]. Biological replicates of parental cells or pooled resistant populations (two different pools for each Pt‐res cell line) were analyzed by 2‐D DIGE proteomic approach performed as previously described [14]. Approximately 1200 protein spots were consistently detected, quantified, normalized, and intergel‐matched in each 2‐D DIGE experiment. In detail, for each condition, we evaluated biological replicates (in quadruplicate) and performed reverse‐labeled by the fluorescent cyanine dyes Cy3 and Cy5. The Cy2 dye was used as the internal standard, generated from an equal combination of all the samples tested in the same experiment, leading to a proper quantitative comparison of proteomic variations with statistical confidence. PCA showed a good experimental reproducibility of 2‐D DIGE results as demonstrated by the close relation between the biological replicates clearly clustering, for each cell model, into two groups corresponding to Pt‐res pool cells (red) vs parental cells (black; Fig. 1A). To be included in the analysis, the differentially expressed protein spots should be detected in 80% of the spot maps (replicates) and should display a statistically significant expression variation of at least 1.3‐fold (*P* < 0.05; Fig. 1A).

Using LC‐MS/MS followed by MASCOT search, we identified 23 of the 26 differentially expressed spots according to our statistics in TOV‐112D; 20/23 in MDAH‐2774 (hereafter MDAH) and 23/27 in OVSAHO cells (Tables S1‐S3). Protein identification resulted the same for master spot numbers 509 and 513 (Ezrin‐EZRI; Tables S1), 292, 320, and 323 (mitochondrial inner membrane protein‐IMMT), 822 and 838 (protein disulfide‐isomerase‐PDIA1), 886 and 887 (protein disulfide‐isomerase A3‐PDIA3; Tables S3) and all likely related to post‐translation modification variables. Indeed, MASCOT search confirmed that the above‐specified proteins present putatively phosphorylated sites. The identified proteins were classified according to the literature or public databases including UniProtKB (http://www.uniprot.org) in different functional classes, as depicted in the pie charts (Fig. 1B).

Among the three EOC Pt‐res/parental models, we did not identify any common differentially expressed protein. However, several identified proteins belonged to the same family or shared common functional classes (Fig. 1B), and eight of them were differentially expressed in at least two cellular models: HSP7C, HNRPL, PHB, prelamin‐A/C (LMNA), ATP synthase subunit, mitochondrial (ATPA), elongation factor Tu mitochondrial (EFTU), heterogeneous nuclear ribonucleoprotein K, and D‐3‐phosphoglycerate dehydrogenase (highlighted in gray in Tables S1‐S3). The different expression of HSP7C, HNRPL, PHB, and LMNA was reported as 3D view spot quantification and validated by western blot with a high concordance between the two analyses (Fig. 1C).

Same results were obtained for differentially expressed proteins specifically altered in each cell model, overall confirming the quality and strength of the used proteomic approach (Fig. S1).

### 3.2 HSP90 is a central hub of the differential expressed proteins network in Pt‐resistant EOC cells

**Fig. 2:**
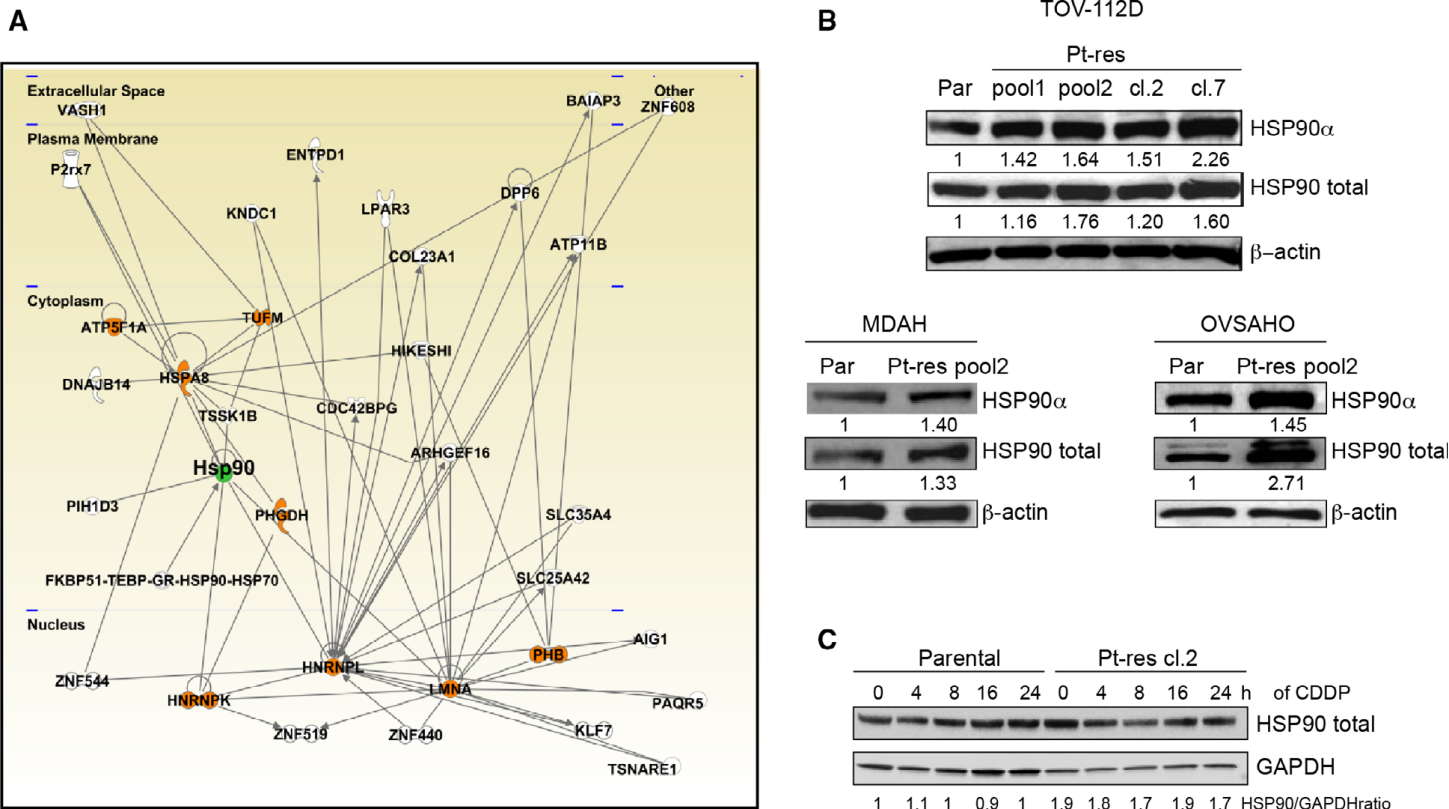
Network of differentially expressed proteins identifying HSP90 as a central hub up‐regulated in CDDP‐resistant cells. (A) IPA was performed looking for direct interactions within a network including the eight proteins differentially expressed by at least two cell lines (colored in orange), with HSP90 emerging as the main hub (colored in green). (B) Western blot analysis revealed an up‐regulation of HSP90α and total HSP90 in CDDP‐resistant TOV‐112D, MDAH, and OVSAHO cells. β‐Actin was used as loading control. Western blot quantification was performed by imagej software. (C) Western blot analysis evaluating the expression of HSP90 in TOV‐112D parental and Pt‐res cl.2 cells treated with CDDP 25 μm for the indicated time points. Densitometric analysis of HSP90 expression (normalized to GAPDH as loading control) is reported under the blot.

Next, to recognize the regulatory pathways in which the identified proteins were involved and hence to elucidate their biological functions and relationship with the Pt resistance, we interrogated the ipa software. We first searched for direct interactions and restricted the analysis to the eight proteins shared by at least two cellular models. Significantly, all eight proteins are associated in a single pathway related with cancer, and the chaperone HSP90 emerged as a relevant central hub in the network (Fig. 2A). Moreover, the IPA analysis also showed a relevant relationship between almost all the identified proteins in each EOC Pt‐res model with 21/22 of the identified proteins closely clustering together in TOV‐112D (Fig. S2A), 20/20 in MDAH (Fig. S2B), and 19/19 in OVSAHO model (Fig. S2C). Interestingly, HSP90 emerged again as a main hub in all the three different networks. Furthermore, when we included all the proteins identified in the three cellular models, HSP90 maintained the role of a central hub in a single network (Fig. S2D).

Based on these results, we next evaluated the expression of total HSP90α in parental and Pt‐res models showing their increased expression in all tested pools (Fig. 2B). Moreover, using two additional TOV‐112D Pt‐res clones (clones #2 and #7) recently generated [7, 8] we further confirmed HSP90 overexpression (Fig. 2B).

Conversely, the mRNA expression of both HSP90α and HSP90β isoforms was not consistently changed in Pt‐res cells, suggesting that HSP90 expression is mainly regulated at post‐transcriptional level (Fig. S3). Moreover, HSP90 expression is not modulated by acute CDDP treatment neither in parental nor in Pt‐res cells (Fig. 2C).

### 3.3 HSP90 inhibitors potentiate CDDP antitumor effect and reverse Pt resistance

**Fig. 3:**
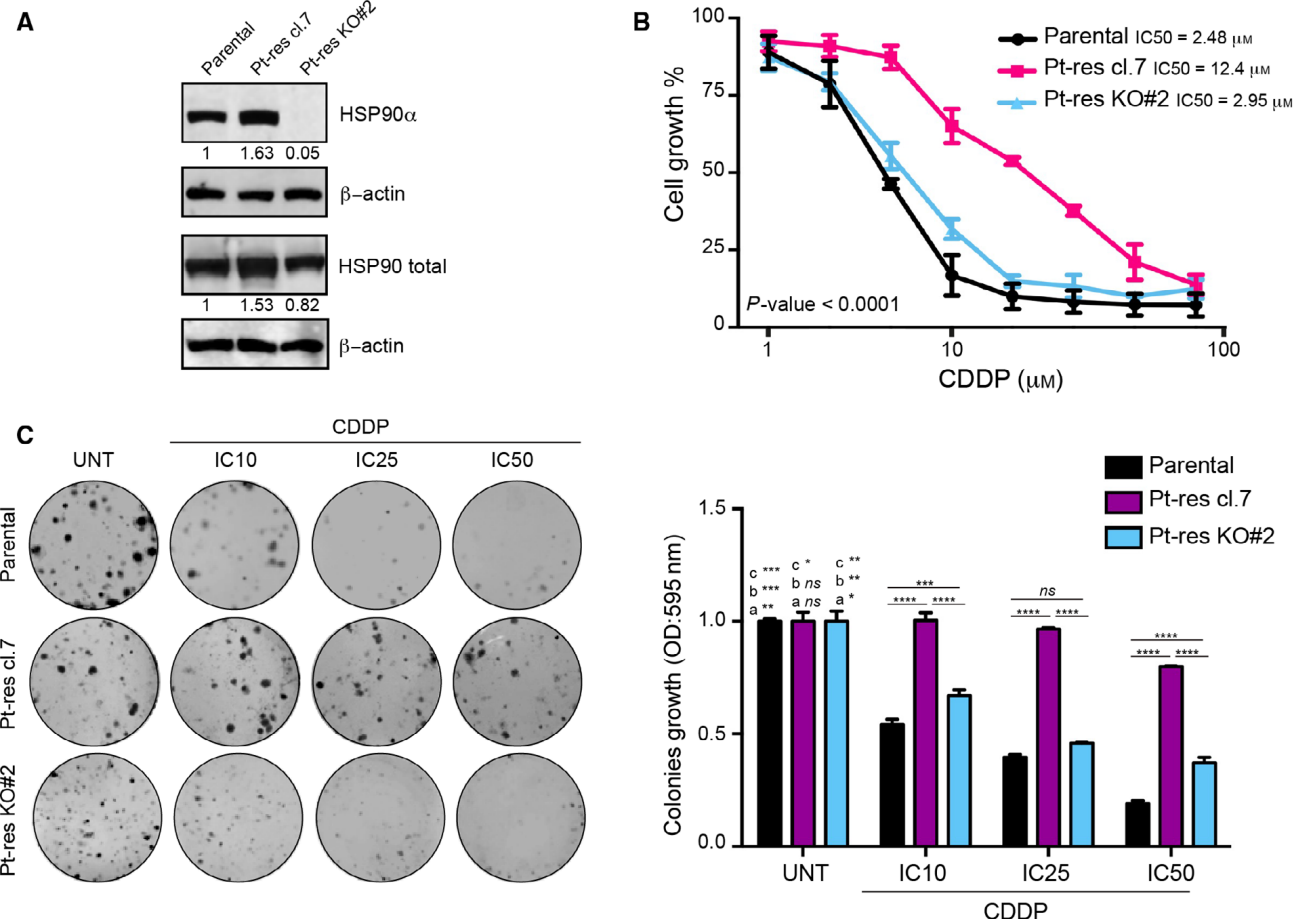
HSP90α knockout sensitizes Pt‐res TOV‐112D cells to CDDP treatment. (A) Western blot analysis evaluating the expression of HSP90α and HSP90 total in TOV‐112D parental, Pt‐res cl.7, and HSP90α knockout Pt‐res KO#2 cells. β‐Actin was used as loading control. Western blot quantification was performed by imagej software. (B) TOV‐112D parental, Pt‐res cl.7, and HSP90α knockout Pt‐res KO#2 cells were treated for 96 h with increasing concentrations of CDDP. Cell growth expressed as percentage of control was assessed by sulforhodamine B colorimetric assay (see Materials and methods). Statistically significant results are reported (*P* < 0.0001). C, Clonogenic assay of TOV‐112D parental, Pt‐res cl.7, and knockout Pt‐res KO#2 cells treated with CDDP at the ${\text{IC}}_{10}^{72\;h}$, ${\text{IC}}_{25}^{72\;h}$, and ${\text{IC}}_{50}^{72\;h}$ doses for parental cells. Representative data of at least three independent experiments performed in triplicates. Statistically significant results calculated with one‐way ANOVA test are reported (a indicates control group, b indicates Pt‐res cl.7 CDDP‐treated cells, and c indicates Pt‐res KO#2 CDDP‐treated cells *P < 0.05, **P < 0.01, ***P < 0.001, and ****P < 0.0001, ns, not statistically significant).

The high levels of HSP90, and particularly of HSP90α, in Pt‐res relative to parental cells suggested that this chaperone protein might represent a potential therapeutic target in Pt‐res EOC setting. In order to test this hypothesis, taking advantage of CRISPR‐Cas9 system, we generated knockout HSP90α cells (Pt‐res KO#2) from TOV‐112D Pt‐res clone #7 (Fig. 3A). HSP90α knockout completely reversed CDDP resistance, as demonstrated by colorimetric cytotoxicity (Fig. 3B) or colony assay (Fig. 3C). Next, we investigated two HSP90i, 17AAG and ganetespib, both in advanced clinical studies. As single agents, ganetespib showed a greater antiproliferative potency than 17AAG, being effective on the majority of the examined cells with IC_50_ values in the low nanomolar range (Table S4). Interestingly, in the majority of the cases, Pt‐res cancer cells were also more resistant to both the HSP90i compared to parental controls.

We then investigated the combination of CDDP with 17AAG (Table S5) or ganetespib (Tables 1 and S6) using equipotent doses (50 : 50 cytotoxic ratio) or an excess of either agent (25 : 75 or 75 : 25 cytotoxic ratio) and evaluated the CI values calculated at 50% (CI_50_) or 75% (CI_75_) of cell lethality, in TOV‐112D and MDAH parental cells and in Pt‐res pools or single clones. By considering strong synergism CI ≤ 0.8, synergism CI ≤ 0.9, additivity CI > 0.9 and ≤ 1.1, and antagonism CI > 1.1 [15, 16], we obtained consistent synergistic antiproliferative effects at equipotent doses and with an excess of HSP90i, in both parental and resistant cells, with slight better results observed by combining CDDP with ganetespib, the latter being effective even at lower doses in combination treatments.

**Table 1:** CI and DRI values for ganetespib and CDDP combination accordingly to different treatment schedules.

Cell lines	GANETESPIB‐CDDP					GANETESPIB→CDDP					CDDP→GANETESPIB				
	CI50 ± SD	CI75 ± SD	CI90 ± SD	DRI50 ± SD		CI50 ± SD	CI75 ± SD	CI90 ± SD	DRI50 ± SD		CI50 ± SD	CI75 ± SD	CI90 ± SD	DRI50 ± SD	
				GANE	CDDP				GANE	CDDP				GANE	CDDP
TOV‐112D	**0.55** ± **0.02**	**0.57** ± **0.06**	**0.61** ± **0.10**	6.02 ± 1.93	2.74 ± 0.39	**0.70** ± **0.04**	**0.78** ± **0.08**	**0.82** ± **0.01**	3.15 ± 0.79	3.09 ± 0.07	**0.68** ± **0.05**	**0.65** ± **0.03**	**0.60** ± **0.04**	5.05 ± 0.14	1.76 ± 0.18
TOV‐112D Pt‐res pool 2	**0.64** ± **0.04**	**0.62** ± **0.07**	**0.66** ± **0.06**	3.67 ± 0.77	1.93 ± 0.33	**0.80** ± **0.09**	**0.83** ± **0.07**	**0.85** ± **0.05**	2.74 ± 0.79	2.89 ± 0.36	**0.72** ± **0.07**	**0.72** ± **0.12**	**0.79** ± **0.09**	3.91 ± 0.11	1.73 ± 0.21
TOV‐112D Pt‐res cl. #2	**0.77** ± **0.07**	**0.86** ± **0.004**	0.74 ± 0.19	2.95 ± 0.23	2.34 ± 0.40	**0.56** ± **0.19**	**0.59** ± **0.16**	**0.65** ± **0.14**	3.24 ± 0.92	3.19 ± 1.07	0.92 ± 0.04	**0.81** ± **0.03**	**0.77** ± **0.04**	2.13 ± 0.64	1.19 ± 0.17
TOV‐112D Pt‐res cl. #7	**0.63** ± **0.14**	**0.60** ± **0.10**	**0.60** ± **0.07**	2.75 ± 0.18	2.29 ± 0.86	0.93 ± 0.02	0.90 ± 0.04	0.73 ± 0.18	2.02 ± 0.22	2.25 ± 0.36	0.81 ± 0.14	**0.81** ± **0.07**	**0.75** ± **0.14**	3.79 ± 0.10	1.56 ± 0.30
MDAH	0.87 ± 0.08	0.86 ± 0.07	0.84 ± 0.09	2.81 ± 0.70	1.86 ± 0.10	0.94 ± 0.05	0.94 ± 0.08	0.98 ± 0.07	2.01 ± 0.24	2.44 ± 0.17	**0.86** ± **0.02**	0.83 ± 0.08	0.87 ± 0.08	4.13 ± 0.96	1.38 ± 0.14
MDAH Pt‐res pool 2	**0.61** ± **0.09**	**0.62** ± **0.07**	**0.69** ± **0.08**	2.69 ± 0.43	3.85 ± 0.52	**0.64** ± **0.10**	**0.68** ± **0.02**	**0.74** ± **0.06**	2.46 ± 0.16	4.78 ± 0.48	**0.84** ± **0.03**	**0.82** ± **0.06**	0.95 ± 0.02	3.07 ± 0.07	2.75 ± 0.20
MDAH Pt‐res cl. #42	0.99 ± 0.02	0.94 ± 0.007	0.90 ± 0.02	2.85 ± 0.89	1.61 ± 0.22	1.07 ± 0.007	0.98 ± 0.05	0.91 ± 0.09	1.97 ± 0.52	1.90 ± 0.49	1.18 ± 0.06	1.06 ± 0.03	0.97 ± 0.10	4.62 ± 1.24	1.05 ± 0.13
MDAH Pt‐res cl.#12	**0.79** ± **0.08**	**0.78** ± **0.05**	**0.76** ± **0.02**	2.87 ± 0.59	2.25 ± 0.07	**0.78** ± **0.07**	**0.67** ± **0.05**	**0.58** ± **0.03**	2.19 ± 0.38	3.09 ± 0.04	0.87 ± 0.04	**0.85** ± **0.001**	**0.83** ± **0.04**	4.50 ± 0.62	1.52 ± 0.03

Cell growth assessment was done by sulforhodamine B colorimetric assay (see Materials and methods). CI values (mean ± SD) from at least three separate experiments performed in quadruplicate computed at 50% (CI50), 75% (CI75) and 90% (CI90) of cell kill by calcusyn software (Biosoft). CIs values: smaller than 0.8 indicate strong synergism highlighted in bold; CIs smaller than 0.9 indicate synergism highlighted in bold; additivity between 0.9 and 1.1 or antagonism more than 1.1. Equipotent doses (50 : 50 cytotoxic ratio) of each of the two agents were evaluated after 96h with a simultaneous (CDDP + GANE) or sequential exposure with 24‐h delay to either drug (GANETESPIB→CDDP; CDDP→GANETESPIB ) as described in Materials and methods. DRI values (mean ± SD) from at least three separate experiments performed in quadruplicate) represent the order of magnitude (fold) of dose reduction obtained for IC_50_ (DRI_50_) in combination setting compared with each drug alone.

Strong synergism (CI ≤ 0.8 ± SD), Synergism (CI ≤ 0.9 ± SD), Additivity (CI > 0.9; ≤ 1.1 ± SD), Antagonism (CI > 1.1 ± SD).

The synergistic interaction between 17AAG or ganetespib and CDDP was also confirmed by the evaluation of the DRIs, which represent the order of magnitude (fold) of dose reduction obtained for the IC_50_ (DRI_50_) of each agent in combination vs single drug treatments (Tables 1 and S5‐S6). Significantly, in hTERT‐immortalized human foreskin fibroblast cell line BJ‐hTERT, we observed antagonist effects (Table S6), suggesting a selective synergistic effect of the double combination on tumor cells.

**Fig. 4:**
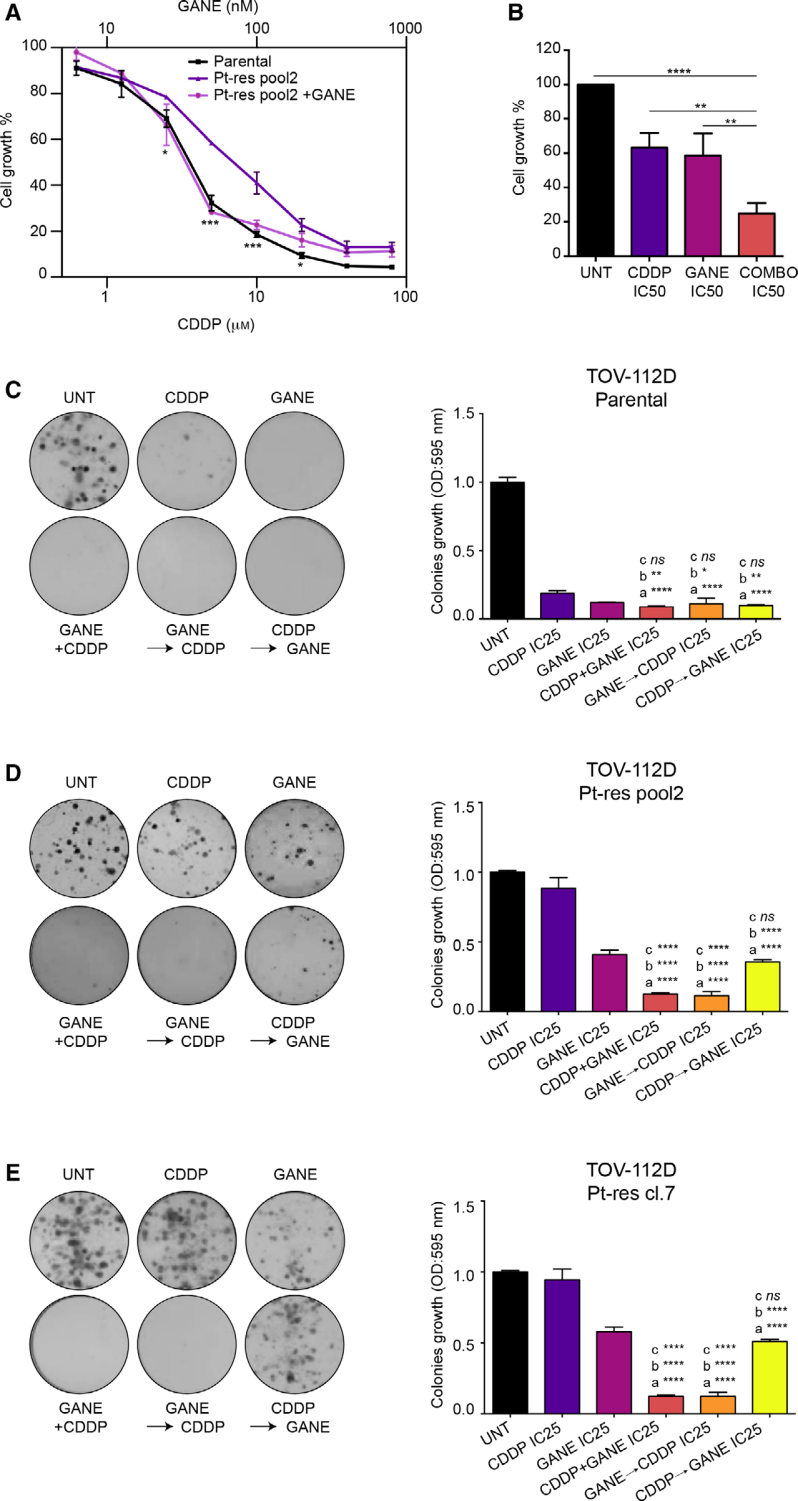
Potentiation of CDDP antitumor effect induced by ganetespib in parental and CDDP‐resistant TOV‐112D cells. (A) TOV‐112D Pt‐res pool 2 cells were treated for 72 h with increasing concentrations of CDDP alone or in combination with ganetespib and compared with TOV‐112D treated with CDDP alone. Cell growth expressed as percentage of control was assessed by sulforhodamine B colorimetric assay (see Materials and methods). (B) Percent (%) of cell growth of TOV‐112D Pt‐res pool 2 cells after 72 h of treatment with CDDP, ganetespib, or CDDP/ganetespib combination, compared to untreated cells. Drugs were used at ${\text{IC}}_{50}^{72\;h}$ doses for parental cells. In A and B, the values are the means ± SD from at least three independent experiments. (C, D, E) Clonogenic assay of TOV‐112D (C) and TOV‐112D Pt‐res cells (D‐E) treated with CDDP, ganetespib, or their combination (simultaneous or sequential exposure with 24 h delay to either drug) at the ${\text{IC}}_{25}^{72\;h}$ doses for parental cells. Representative data of at least three independent experiments performed in triplicates. Statistically significant results calculated with one‐way ANOVA test are reported (a indicates control group, b indicates CDDP‐treated cells, and c indicates ganetespib‐treated cells *P < 0.05, **P < 0.01, ***P < 0.001, and ****P < 0.0001, ns, not statistically significant).

For ganetespib/CDDP combination, we also compared simultaneous *vs* sequential (with 24 h delay between the two agents) treatment, demonstrating better effect in concomitant treatment schedule or when ganetespib preceded CDDP. Notably, in these setting in Pt‐res cells we observed a clear potentiation of CDDP effect with DRI ranging from a minimum of 1.4 up to almost 5 (Table 1). Indeed, concomitant treatment of TOV‐112D Pt‐res cells with ganetespib and CDDP, demonstrated a complete reversion of the CDDP resistance (Fig. 4A) and a significant synergistic reduction of cell viability, compared to either agent alone (Fig. 4B).

Moreover, we demonstrated that concomitant ganetespib/CDDP or sequential treatment of ganetespib 24 h before CDDP, using ${\text{IC}}_{25}^{72\;h}$ of either agent, completely suppressed colony formation in parental (Fig. 4C) and Pt‐res TOV‐112D cells (Fig. 4D,E), compared with single agents, or CDDP treatment preceding ganetespib. This effect was also confirmed at ${\text{IC}}_{10}^{72\;h}$ dosages, particularly in parental cells (Fig. S4A,C).

Furthermore, since TOV‐112D Pt‐res cells were cross‐resistant to doxorubicin [6] (Table S4), another chemotherapeutic drug commonly used to treat EOC patients, we evaluated concomitant treatment of this agent plus ganetespib, also demonstrating synergistic interaction with CI_50_ lower than 0.8 and DRI for doxorubicin up to 5 (Table S7), suggesting that HSP90i may be able to overcome a common mechanism of acquired drug resistance.

**Fig. 5:**
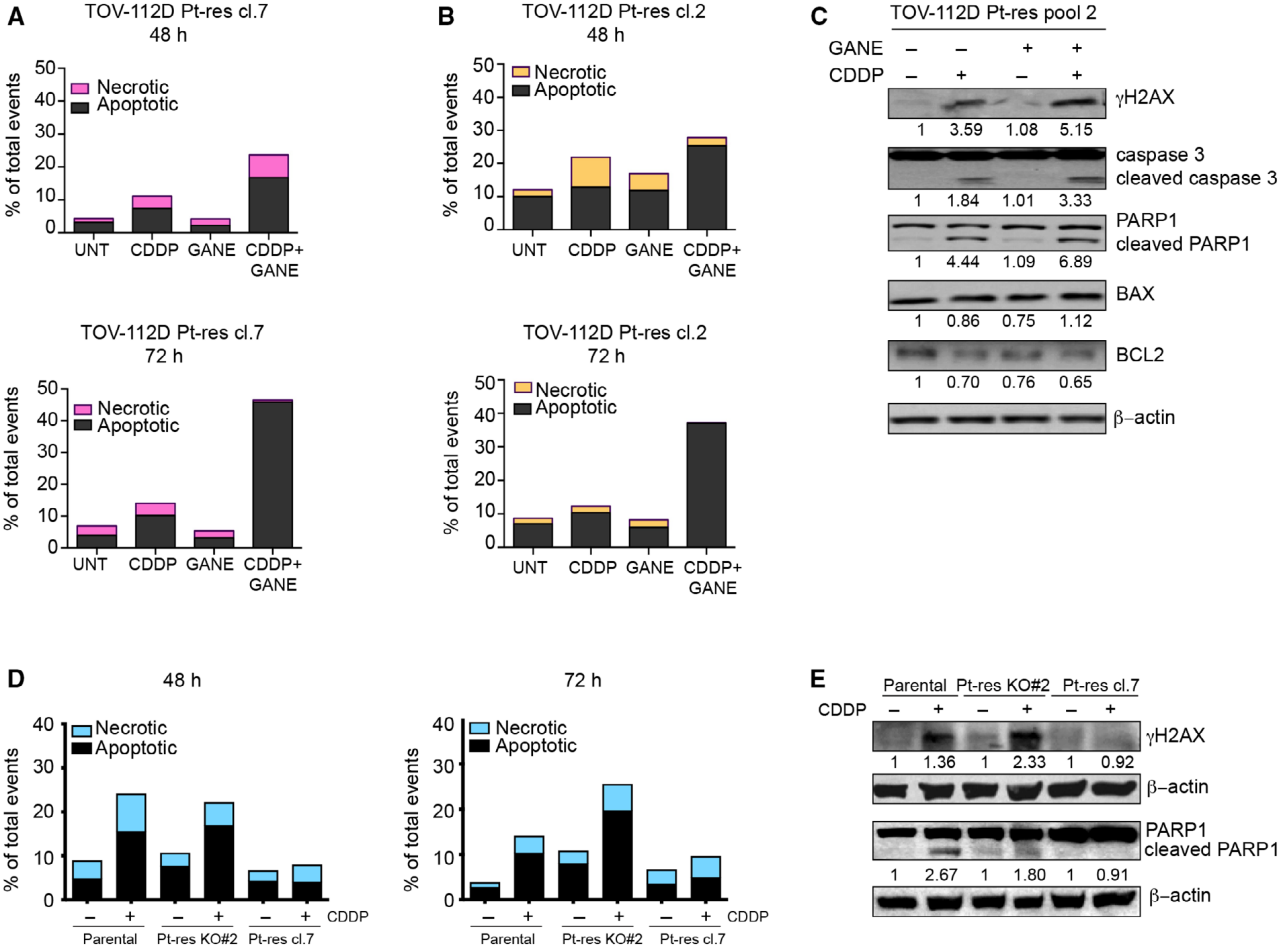
HSP90 pharmacological inhibition or HSP90α knockout increases the pro‐apoptotic and DNA damage effect of CDDP in Pt‐res TOV‐112D cells. (A) Apoptosis and necrosis evaluated by flow cytometry after Annexin V‐FITC and propidium iodide staining in TOV‐112D Pt‐res cl. 7 and (B) cl. 2 cells, untreated or treated for 48 h (upper panels) or 72 h (lower panels), with CDDP and/or ganetespib at ${\text{IC}}_{50}^{72\;h}$ doses of parental cells. (C) Western blot analysis of γH2AX, PARP1 and caspase 3 cleavage, BAX and BCL2 expression, in TOV‐112D Pt‐res pool 2 cells untreated or treated with CDDP and/or ganetespib at the doses indicated above. β‐Actin expression serves as loading control. Western blot quantification was performed by imagej software. (D) Apoptosis and necrosis evaluated by flow cytometry after Annexin V‐FITC and propidium iodide staining in TOV‐112D, TOV‐112D Pt‐res cl. 7 and in HSP90α knockout TOV‐112D Pt‐res KO#2 cells, untreated or treated for 48 or 72 h with CDDP at ${\text{IC}}_{50}^{72\;h}$ doses of parental cells.

Finally, we observed that the potentiation of CDDP antitumor effect and the reversion of Pt resistance by HSP90α knockout or pharmacological inhibitors such as ganetespib was due to an increased apoptosis as demonstrated by the evaluation of Annexin V positivity and of cleaved PARP1 and caspase‐3 expression both in TOV‐112D and in MDAH models (Figs 5A‐E and S5A‐C). The observed increased apoptosis observed was accompanied by a clear induction of γH2AX, indicating increased DNA damage (Figs 5C,E and S5A). These data support the possibility that the intrinsic apoptotic pathway was activated for an increased DNA damage. Accordingly, with respect to control or single‐agent treatments, the ganetespib plus CDDP combination induced a clear up‐regulation of the pro‐apoptotic BAX with a down‐regulation of the antiapoptotic Bcl‐2 protein and an overall increase of the BAX/Bcl‐2 ratio (Fig. 5C). Interestingly, we also observed in TOV‐112D parental and Pt‐res cells a decrease of HSP90α protein expression induced by ganetespib/CDDP combination compared with untreated or single agents‐treated cells (Fig. S6A).

**Fig. 6:**
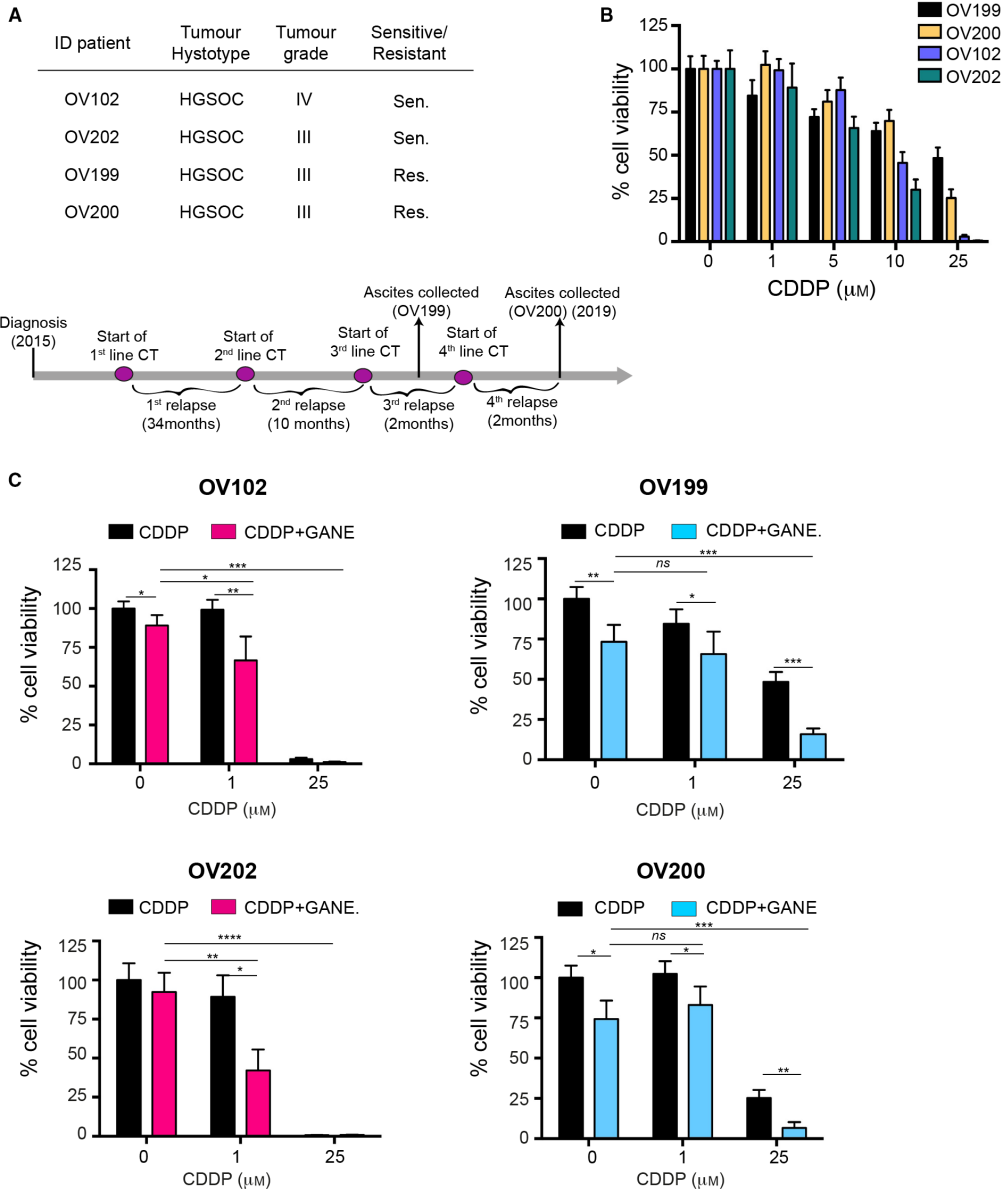
Increased CDDP sensitivity of EOC primary cells by ganetespib. (A) Table reporting the pathological variables of patients included in this study. At the bottom, the timeline showing the dates of diagnosis, chemotherapy treatments, relapses, and ascite collections of the EOC patient from whom OV199 and OV200 primary cultures were established. (B‐C) MTS assay of primary cells established from EOC patient's ascites (described in A), treated for 72 h with increasing doses of CDDP alone (B) or in combination with ganetespib 25 nm (C). Data are expressed as the percentage of viable cells in treated respect to the untreated condition and represent the mean (±SD) of three independent experiments. Statistical significance was determined by a two‐tailed, unpaired Student's *t*‐test (*P < 0.05, **P < 0.01, ***P < 0.001 and ****P < 0.0001, ns, not statistically significant).

The collected data suggested that Pt‐res EOC cells were particularly sensitive to the ganetespib/CDDP. We thus tested this possibility using primary cells obtained from ascites of two Pt‐sensitive EOC patients and from one patient that developed Pt resistance over the time (Fig. 6A). Cultured cells responded to CDDP treatment as predicted by the Pt sensitivity of the corresponding patients (Fig. 6B). The use of ganetespib effectively increased the activity of CDDP in both Pt‐sensitive and Pt‐res primary cultures with some differences. In Pt‐sensitive cells (i.e., OV102 and OV202), ganetespib increased the activity of low doses of CDDP (1 µm) while it had no effects when high doses of CDDP were used (25 µm). In these primary cultures at the dose used (25 nm), ganetespib had no or minor effects. (Fig. 6C left graphs). In Pt‐res cells (i.e., OV199 and OV200), ganetespib used alone significantly reduced cell survival and this activity prevailed on the one of low doses of CDDP (1 µm). Importantly, the addition of ganetespib to a high dose of CDDP (25 µm) significantly improved CDDP efficacy (Fig. 6C right graphs), confirming that the combination therapy is effective also on *ex vivo* cultures of patient‐derived tumor cells.

### 3.4 *In vivo* synergistic antitumor effect of CDDP and ganetespib combination

**Fig. 7:**
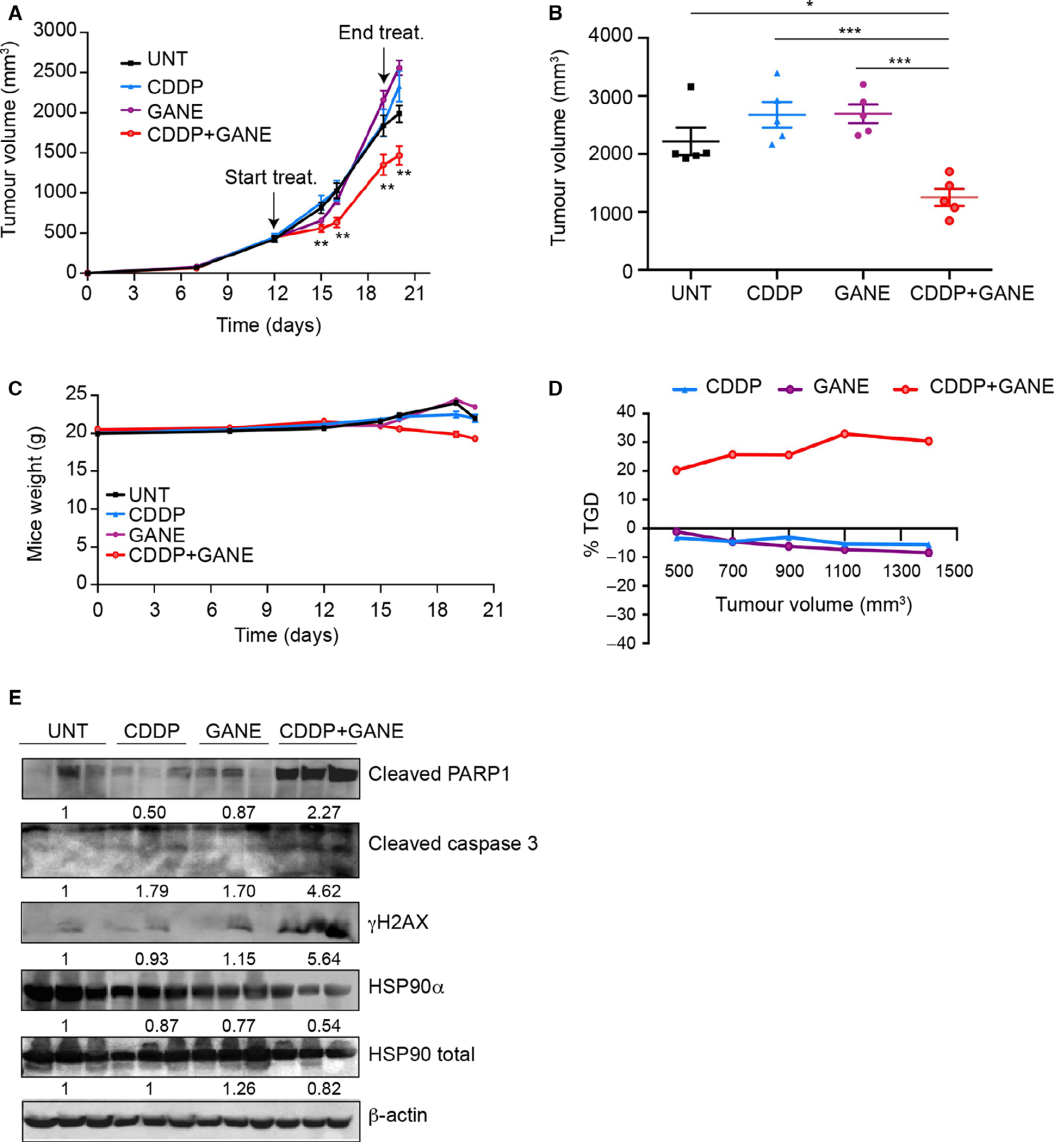
Potentiation of CDDP antitumor effect induced by ganetespib *in vivo* CDDP‐resistant TOV‐112D xenograft model. TOV‐112D Pt‐res pool 2 cells (5 × 10^6^) were s.c. injected into NSG mice as described in Materials and methods. When tumors were established, mice (five/group) were treated once a week for two weeks, with CDDP (2.5 mg·kg^−1^ i.p.), ganetespib (GANE; 75 mg·kg^−1^ i.p.), both drugs in combination, or their respective vehicles (UNT). (A) Relative TV measured at prespecified time points (Means ± SEM). (B) TVs at cutoff when mice were sacrificed. Data are shown as means ± SEM. (C) Mice body weight as surrogate indicator of toxicity for *in vivo* experiment reported in A. Body weight was measured three times/week. (D) TGD, indicating the mean rate of tumor growth in the treatment groups relative to control untreated mice (see Materials and methods). Statistically significant results calculated with one‐way ANOVA test are reported (*P < 0.05, **P < 0.01, and ***P < 0.001). (E) Western blot analysis of γH2AX, cleaved PARP1, cleaved caspase 3, HSP90α, and total HSP90, in lysates from three representative xenograft tumor samples from each treatment group (see Materials and methods). β‐Actin expression serves as loading control. Western blot quantification was performed by imagej software using the mean value of the three samples for each experimental group.

Finally, we used the CDDP/ganetespib combination to treat mice‐bearing xenograft tumors formed by TOV‐112D Pt‐res pool 2 cells. When tumors reached ~ 500 mm^3^ of volume, mice were randomized in four treatment groups: control, CDDP, ganetespib, and CDDP plus ganetespib. Significantly, CDDP/ganetespib‐treated group was the only one that produced a statistically significant TV reduction compared with all other groups (Fig. 7A,B). Tumors in vehicle‐treated, CDDP or ganetespib alone treated mice, grew rapidly and reached the endpoint size within 20 days, when the average TV of single agents‐treated groups was even slightly increased compared with controls. We observed a modest decrease of mice body weight in the combination treatment; however, it was less than 10% and was not paralleled by other signs of acute or delayed toxicity (Fig. 7C).

The synergistic effect of the CDDP/ganetespib combination treatment was confirmed by the resulting TGD that reached a peak of about 35% indicating that the mean rate of tumor growth was more than three‐fold higher in the controls or single agents‐treated groups, than the combination setting (Fig. 7D).

Western blot analyses on tumor lysates demonstrated that the combination treatment induced a clear reduction in HSP90α and of total HSP90 levels that was accompanied by a prominent increase of PARP1 and caspase 3 cleavage and of γH2AX expression, compared with the other groups, confirming *in vivo* what observed *in vitro* (Fig. 7E). Conversely, in xenograft model of parental TOV‐112D cells, by using carboplatin and ganetespib, we showed that both agents were effective in inhibiting tumor growth in monotherapy and that their combination did not produce any clear potentiation of the antitumor effect (Fig. S6B,C).

## 4 Discussion

Despite numerous efforts to develop novel therapeutic approaches, the treatment of refractory/resistant EOC patients after first‐line Pt treatment remains a clinical challenge. Different mechanisms of Pt resistance have been described, including alterations in intracellular concentration due to influx or efflux defects as well as increased DNA repair or survival pathways [17]. Indeed, several attempts have been undertaken to bypass these mechanisms with relative scarce success, mainly due to the multimodal nature of the resistance [17]. The complex and heterogeneous genetic profile of EOC, particularly of HGSOC, limited the stratification of patients subtypes also preventing the identification of distinct genetic features predicting Pt resistance [5].

In the present study, by using a proteomic approach followed by a bioinformatic analysis using IPA software, we demonstrated that HSP90 emerged as a central hub in a protein network, associated with ‘cancer’, connecting the identified differentially expressed proteins in all three Pt‐res models, compared with parental controls. HSP90 was overexpressed in Pt‐res compared to parental cells, mostly due to HSP90α isoform protein overexpression, further suggesting a potential role in the mechanism of resistance. Indeed, we demonstrated that HSP90α knockout sensitizes Pt‐res cells to CDDP treatment. Most importantly, by targeting HSP90 with specific HSP90i, we demonstrated, *in vitro, ex vivo* in primary cultures derived from the ascites of Pt‐res EOC patients and *in vivo* in Pt‐res EOC xenograft models, synergistic antitumor effect in combination with CDDP.

Stress‐associated phenotype, including proteotoxic stress, recently emerged as an additional hallmark of cancer, also highlighting the critical role in cancer cells of chaperons such as HSP90 that is involved in protein homeostasis [18]. HSP90 is a family of ATP‐dependent molecular chaperones that with the help of co‐chaperones modulate signal transduction and stress responses by regulating the folding, stability, and activity of client proteins, including several oncogenic molecules (i.e., HER2, estrogen receptors, AKT, MET, VEGFR, BRCA‐1, MMP2) [19]. Thereby, HSP90 is also involved in many other hallmarks of cancer, including cell growth, survival, angiogenesis, metastases, and immune response [19, 20]. Indeed, high HSP90 expression levels have been reported in many tumors and correlated with metastasis status and chemo‐resistance as well as recently also with immune refractory status [19, 20, 21]. Overexpression of HSP90 has been also reported in ovarian carcinoma where it was linked to the FIGO stage of the disease [22]. Moreover, by using an ovarian cancer expression library for a serological screening, HSP90 emerged as a specific tumor antigen for this disease [22].

Notably, the two largest groups of identified proteins in all three Pt‐res EOC models were those under the functional clusters ‘stress response, chaperones and protein fate’ and ‘nucleic acid processing and DNA damage’. An additional functional cluster of identified proteins is EMT, cancer stem cells, and cytoskeleton organization”, and included common biological features we have previously attributed to our Pt‐res EOC models [6]. Interestingly, numerous identified proteins and particularly the eight deregulated in at least two Pt‐res models have been directly connected with Pt resistance and/or ovarian cancer aggressive features [23, 24, 25, 26, 27]. For example, among those proteins overexpressed in our PT‐res EOC models: molecular chaperones, including HSP90 or proteins involved in drug detoxification such as peroxiredoxins, (PRDX) were reported up‐regulated in PT‐res cancer cells and/or induced by CDDP treatment [23]; similarly, PRDX2, the chaperone GRP75, and the protein involved in stress response EFTU were proposed as candidate biomarkers of drug‐resistant disease in ovarian cancers [24]; furthermore, PHB, involved in cancer proliferation and metastasis, and PRDX4 were significantly found increased in ovarian cancer tissues from chemotherapy nonresponders patients [27]. Conversely, among those proteins whose expression we have found reduced in Pt‐res models: loss of nuclear lamina protein LMNA in EOC was associated with metastasis and poor prognosis [25], while reduced secreted expression of the dehydrogenase 3‐PGDH was identified among candidate circulating CDDP‐resistant biomarkers from EOC cell secretomes [26].

In mammalian cells, there are two major HSP90 isoforms that are the constitutively expressed: HSP90β and the stress‐inducible HSP90α, sharing a conserved ATP‐binding domain targeted by most HSP90i [28, 29].

HSP90α protein is expressed at differing levels in a tissue‐specific manner and is specifically up‐regulated in several cancers, while the corresponding HSP90AA1 gene is not altered in the majority of tumors [28]. In line with our data, it was previously demonstrated that overexpression of HSP90α in ovarian cancer cells decreased the chemosensitivity to CDDP [30].

Interestingly, recently it was pointed out that tumor addiction to a chaperon like HSP90 is due to its level of connectivity with other chaperons and/or to the hub role played within a network of proteins crucial for cancer cells homeostasis, rather than to the overexpression of the protein itself [31]. In other words, due to the dynamic nature of the binding with client proteins in order to modulate the plasticity of signaling pathways, only when HSP90 is hyper‐connected and fully integrated with client proteins and chaperone machinery, then its targeting with specific inhibitors is therapeutically effective [31]. Indeed, a correlation between HSP90 isoform levels and HSP90i sensitivity in cancer cells is debated [32]. On this regard, although we found in all tested Pt‐res cells HSP90α and total HSP90 protein overexpression compared with controls, HSP90 was not identified by the proteomic study as differentially expressed, probably because the magnitude of level change was not prominent and/or homogenous. Yet, we propose that the hub role of HSP90 highlighted in Pt‐res cells was more biologically relevant than the mere protein overexpression and might explain the clear synergistic interaction we observed between HSP90i and CDDP in Pt‐res EOC cells. Interestingly, data‐mining meta‐analyses integrating the results from multiple siRNA screening in EOC also identify HSP90 as a central hub and an effective therapeutic target [33]. Notably, we have also discovered HSP90 as a critical hub within a network of differentially expressed proteins identified in a Pt‐res NSCLC isogenic model [9], supporting the possibility that the same mechanism is present in different tumor types. Indeed preliminary results showed also in this Pt‐res NSCLC model a synergistic antitumor interaction between HSP90i and CDDP^1^.

Classical HSP90i act by competitively binding the ATP‐binding site of HSP90, consequently halting the progression of the protein folding machinery, leading to the degradation of most client proteins [34]. Geldanamycin (GA), a large naturally occurring compound and its ansamycin derivatives 17AAG and 17DMAG were the first HSP90i to be tested [35, 36]. In accord with our observation previous *in vitro* studies reported that the combination of GA, 17DMAG, or 17AAG with CDDP remarkably inhibited cell proliferation and partially reverse the drug resistance of CDDP‐resistant SKOV‐3 ovarian cancer cells [37]. Here we add the new notions that ganetespib is more effective than 17AAG in improving CDDP efficacy and prove for the first time that the combination therapy is active *in vivo* in Pt‐res ovarian cancer models and in primary ovarian cancer cells derived from the ascites of Pt‐res ovarian cancer patients. Preclinical studies have shown that ganetespib has greater potency than first‐generation inhibitors such as 17AAG in several cancers [38, 39, 40] also in combination therapy (i.e., carboplatin, taxanes, and etoposide), with a better therapeutic index and no evidence of the cardiac, ocular or liver toxicity observed with other HSP90i, thus emerging as a promising anticancer agent being tested in several clinical trials [38, 41, 42, 43]. These data are in accord with our observation that ganetespib was able to revert the cross‐resistance of Pt‐res cells to doxorubicin, confirming the critical role of HSP90 in conferring a complex prosurvival chemo‐resistant phenotype, likely by regulating tumor apoptosis. Of note, the lack of synergistic interaction between ganetespib and CDDP observed in normal human fibroblasts suggested a selective action on tumor cells and thus a good therapeutic index of the combined approach.

Our data support the possibility that the multimodal mechanisms of Pt resistance acquisition in our three EOC cell models, that differ in histology and genetic backgrounds, are commonly dependent on the addiction to HSP90 hub function, thereby rendering resistant cells particularly vulnerable to the combined HSP90i/CDDP approach. Interestingly, the lack of a clear synergistic interaction *in vivo* xenograft parental EOC models of ganetespib plus Pt compound as compared with results obtained in Pt‐res cells, confirmed, at least in part, this hypothesis. Anyhow to our knowledge, this study is the first to show the reversion of CDDP resistance and the synergistic interaction of ganetespib with CDDP, both *in vitro* and *in vivo* in Pt‐res ovarian cancer models, providing a rationale to clinically explore this combination to rechallenge refractory/resistant EOC patients.

In unselected cancer patients, the clinical benefit of HSP90i has been modest so far, however in selected populations, with distinct molecular features and/or in refractory/resistant subgroups a promising activity has been shown [44, 45]. Ganetespib has been evaluated in combination with weekly paclitaxel in Pt‐res ovarian cancer patients and was generally well‐tolerated with no DLTs observed [46]; however, no survival benefit was demonstrated in a randomized phase 2 trial evaluating this combination regimen vs paclitaxel alone [47]. Notably, a recent designed randomized multiarms phase 2 study, not yet recruiting, will evaluate the potential of ganetespib in combination with carboplatin in Pt‐sensitive ovarian cancer patients (relapse > 6 months after previous Pt‐based treatment) *vs* carboplatin plus paclitaxel or plus gemcitabine^3^.

In our opinion, strategies to identify HSP90 addiction within tumor cells could help in patient’s stratification/selection. In this regard, changes in the expression levels, the composition of chaperone complex, or the level of interaction with other chaperones/clients, as well the cellular location of HSP90, could be considered. Interestingly, tumor cells constitutively secrete HSP90 and recent studies showed that the plasma level of HSP90α correlates with the pathologic stage of cancer in patients, suggesting the potential of circulating HSP90 as predictive biomarker [29, 48]. Similarly, a gene expression classifier that predicts sensitivity to HSP90i in a subgroup ovarian and breast cultured cells, cell line‐derived xenografts, and PDX models, was recently generated and could be explored in the frame of translational clinical studies aiming at testing the feasibility and efficacy of HSP90i plus Pt in Pt‐res EOC patients [32].

## 5 Conclusion

In conclusion in this study starting from an unbiased proteomic approach, we identified HSP90 as a central druggable target in Pt‐res EOC cells. We then showed that the HSP90i ganetespib has a strong synergistic effect with CDDP in Pt‐res EOC cells *in vitro*, *ex vivo*, and *in vivo*. Since the association between ganetespib and chemotherapy had an acceptable safety profile in cancer patients, this study has an immediate translational relevance and provides a strong rationale of an effective therapeutic strategy for the treatment of Pt‐res EOC patients for whom we still do not have valid therapeutic options.

## Conflict of interest

The authors declare no conflict of interest.

## Author contributions

RL, MS, and BP all contributed to conceptualization, investigation, development of methodology, writing—original draft; LA, FI, FC, MSC, MRM, TM, LA, AC contributed to investigation, development of methodology, validation; EDG and FB contributed to conceptualization, development of methodology, writing—review and editing; GB contributed to conceptualization, writing—review and editing, supervision, funding acquisition; AB contributed to conceptualization, writing—original draft, writing—review and editing, supervision, funding acquisition.

### Peer Review

The peer review history for this article is available at https://publons.com/publon/10.1002/1878‐0261.12883.

## Supporting information


**Fig. S1**. Validation by Western blot of protein identified in the cellular models as differentially expressed in the 2‐D DIGE LC‐MS/MS analysis.
**Fig. S2**. Ingenuity Pathway Analysis of all identified proteins.
**Fig. S3**. mRNA expression in parental and Pt‐res EOC cell models.
**Fig. S4**. Clonogenic assay of TOV‐112D and TOV‐112D Pt‐res cells.
**Fig. S5**. Pro‐apoptotic effect of CDDP and/or ganetespib in Pt‐ MDAH Pt‐res and in TOV‐112D parental cells.
**Fig. S6.** Effect of CDDP and/or ganetespib on HSP90α expression in TOV‐112D and TOV‐112D Pt‐res cells and on tumor growth of TOV‐112D parental cells xenograft model.

**Table S1.** Differentially expressed proteins identified by mass spectrometry in TOV‐112D model.
**Table S2**. Differentially expressed proteins identified by mass spectrometry in MDAH model.
**Table S3**. Differentially expressed proteins identified by mass spectrometry in OVSAHO model.

**Table S4.** Sensitivity of EOC cell lines to single agent treatments.

**Table S5**. Combination index (CI) and dose reduction index (DRI) values for 17AAG and CDDP combination treatment accordingly to different cytotoxic ratio.
**Table S6**. Combination index (CI) and dose reduction index (DRI) values for ganetespib and CDDP combination treatment accordingly to different cytotoxic ratio.
**Table S7**. Combination index (CI) and dose reduction index (DRI) values for ganetespib and doxorubicin combination treatment at equitoxic ratio.

## Footnotes

^2^
https://clinicaltrials.gov/ct2/results?cond=cancer&term=hsp90+inhibitor&cntry=&state=&city=&dist=. In. 2020.

^1^ Lombardi R., Pucci B, Addi L and Budillon A, unpublished observations.

^3^
https://clinicaltrials.gov/ct2/show/NCT03783949?term=ganetespib&cond=Ovary+Cancer&draw=2&rank=1. In. 2020.

## References

[1] Reid BM , Permuth JB & Sellers TA (2017) Epidemiology of ovarian cancer: a review. Cancer Biol Med 14, 9–32. 28443200 10.20892/j.issn.2095-3941.2016.0084 PMC5365187

[2] Colombo N , Sessa C , du Bois A , Ledermann J , McCluggage WG , McNeish I , Morice P , Pignata S , Ray‐Coquard I , Vergote I *et al*. (2019) ESMO‐ESGO consensus conference recommendations on ovarian cancer: pathology and molecular biology, early and advanced stages, borderline tumours and recurrent disease dagger. Ann Oncol 30, 672–705. 31046081 10.1093/annonc/mdz062

[3] Lheureux S , Gourley C , Vergote I & Oza AM (2019) Epithelial ovarian cancer. Lancet 393, 1240–1253. 30910306 10.1016/S0140-6736(18)32552-2

[4] Damia G & Broggini M (2019) Platinum resistance in ovarian cancer: role of DNA repair. Cancers 11, 119. 10.3390/cancers11010119 PMC6357127 30669514

[5] Kim S , Han Y , Kim SI , Kim HS , Kim SJ & Song YS (2018) Tumor evolution and chemoresistance in ovarian cancer. NPJ Precis Oncol 2, 20. 30246154 10.1038/s41698-018-0063-0 PMC6141595

[6] Sonego M , Pellizzari I , Dall'Acqua A , Pivetta E , Lorenzon I , Benevol S , Bomben R , Spessotto P , Sorio R , Gattei V *et al*. (2017) Common biological phenotypes characterize the acquisition of platinum‐resistance in epithelial ovarian cancer cells. Sci Rep 7, 7104. 28769043 10.1038/s41598-017-07005-1 PMC5540908

[7] Sonego M , Poletto E , Pivetta E , Nicoloso MS , Pellicani R , Vinciguerra GLR , Citron F , Sorio R , Mongiat M & Baldassarre G (2019) TIMP‐1 is overexpressed and secreted by platinum resistant epithelial ovarian cancer cells. Cells 9, 6. 10.3390/cells9010006 PMC7016675 31861382

[8] Lorenzon I , Pellarin I , Pellizzari I , D'Andrea S , Belletti B , Sonego M , Baldassarre G & Schiappacassi M (2019) Identification and characterization of a new platinum‐induced TP53 mutation in MDAH ovarian cancer cells. Cells 9 , 36. 10.3390/cells9010036 PMC7016977 31877751

[9] Milone MR , Lombardi R , Roca MS , Bruzzese F , Addi L , Pucci B & Budillon A (2019) Novel pathways involved in cisplatin resistance identified by a proteomics approach in non‐small‐cell lung cancer cells. J Cell Physiol 234, 9077–9092. 30362533 10.1002/jcp.27585

[10] Alfano L , Caporaso A , Altieri A , Dell'Aquila M , Landi C , Bini L , Pentimalli F & Giordano A (2019) Depletion of the RNA binding protein HNRNPD impairs homologous recombination by inhibiting DNA‐end resection and inducing R‐loop accumulation. Nucleic Acids Res 47, 4068–4085. 30799487 10.1093/nar/gkz076 PMC6486545

[11] Ran FA , Hsu PD , Wright J , Agarwala V , Scott DA & Zhang F (2013) Genome engineering using the CRISPR‐Cas9 system. Nat Protoc 8, 2281–2308. 24157548 10.1038/nprot.2013.143 PMC3969860

[12] Bruzzese F , Pucci B , Milone MR , Ciardiello C , Franco R , Chianese MI , Rocco M , Di Gennaro E , Leone A , Luciano A *et al*. (2013) Panobinostat synergizes with zoledronic acid in prostate cancer and multiple myeloma models by increasing ROS and modulating mevalonate and p38‐MAPK pathways. Cell Death Dis 4, e878. 24157872 10.1038/cddis.2013.406 PMC3920938

[13] Terranova‐Barberio M , Roca MS , Zotti AI , Leone A , Bruzzese F , Vitagliano C , Scogliamiglio G , Russo D , D'Angelo G , Franco R *et al*. (2016) Valproic acid potentiates the anticancer activity of capecitabine *in vitro* and *in vivo* in breast cancer models via induction of thymidine phosphorylase expression. Oncotarget 7, 7715–7731. 26735339 10.18632/oncotarget.6802 PMC4884949

[14] Milone MR , Pucci B , Bifulco K , Iannelli F , Lombardi R , Ciardiello C , Bruzzese F , Carriero MV & Budillon A (2015) Proteomic analysis of zoledronic‐acid resistant prostate cancer cells unveils novel pathways characterizing an invasive phenotype. Oncotarget 6, 5324–5341. 25481874 10.18632/oncotarget.2694 PMC4467152

[15] Chou TC & Talalay P (1984) Quantitative analysis of dose‐effect relationships: the combined effects of multiple drugs or enzyme inhibitors. Adv Enzyme Regul 22, 27–55. 6382953 10.1016/0065-2571(84)90007-4

[16] Piro G , Roca MS , Bruzzese F , Carbone C , Iannelli F , Leone A , Volpe MG , Budillon A & Di Gennaro E (2019) Vorinostat potentiates 5‐fluorouracil/cisplatin combination by inhibiting chemotherapy‐induced EGFR nuclear translocation and increasing cisplatin uptake. Mol Cancer Ther 18, 1405–1417. 31189612 10.1158/1535-7163.MCT-18-1117

[17] Galluzzi L , Vitale I , Michels J , Brenner C , Szabadkai G , Harel‐Bellan A , Castedo M & Kroemer G (2014) Systems biology of cisplatin resistance: past, present and future. Cell Death Dis 5, e1257. 24874729 10.1038/cddis.2013.428 PMC4047912

[18] Guang MHZ , Kavanagh EL , Dunne LP , Dowling P , Zhang L , Lindsay S , Bazou D , Goh CY , Hanley C , Bianchi G *et al*. (2019) Targeting proteotoxic stress in cancer: a review of the role that protein quality control pathways play in oncogenesis. Cancers 11, 66. 10.3390/cancers11010066 PMC6356294 30634515

[19] Schopf FH , Biebl MM & Buchner J (2017) The HSP90 chaperone machinery. Nat Rev Mol Cell Biol 18, 345–360. 28429788 10.1038/nrm.2017.20

[20] Trepel J , Mollapour M , Giaccone G & Neckers L (2010) Targeting the dynamic HSP90 complex in cancer. Nat Rev Cancer 10, 537–549. 20651736 10.1038/nrc2887 PMC6778733

[21] Song KH , Oh SJ , Kim S , Cho H , Lee HJ , Song JS , Chung JY , Cho E , Lee J , Jeon S *et al*. (2020) HSP90A inhibition promotes anti‐tumor immunity by reversing multi‐modal resistance and stem‐like property of immune‐refractory tumors. Nat Commun 11, 562. 31992715 10.1038/s41467-019-14259-y PMC6987099

[22] Hoter A & Naim HY (2019) Heat shock proteins and ovarian cancer: important roles and therapeutic opportunities. Cancers 11, 1389. 10.3390/cancers11091389 PMC6769485 31540420

[23] Castagna A , Antonioli P , Astner H , Hamdan M , Righetti SC , Perego P , Zunino F & Righetti PG (2004) A proteomic approach to cisplatin resistance in the cervix squamous cell carcinoma cell line A431. Proteomics 4, 3246–3267. 15378690 10.1002/pmic.200400835

[24] Cruz IN , Coley HM , Kramer HB , Madhuri TK , Safuwan NA , Angelino AR & Yang M (2017) Proteomics analysis of ovarian cancer cell lines and tissues reveals drug resistance‐associated proteins. Cancer Genomics Proteomics 14, 35–51. 28031236 10.21873/cgp.20017 PMC5267499

[25] Gong G , Chen P , Li L , Tan H , Zhou J , Zhou Y , Yang X & Wu X (2015) Loss of lamin A but not lamin C expression in epithelial ovarian cancer cells is associated with metastasis and poor prognosis. Pathol Res Pract 211, 175–182. 25499720 10.1016/j.prp.2014.11.008

[26] Teng PN , Wang G , Hood BL , Conrads KA , Hamilton CA , Maxwell GL , Darcy KM & Conrads TP (2014) Identification of candidate circulating cisplatin‐resistant biomarkers from epithelial ovarian carcinoma cell secretomes. Br J Cancer 110, 123–132. 24178762 10.1038/bjc.2013.687 PMC3887292

[27] Sehrawat U , Pokhriyal R , Gupta AK , Hariprasad R , Khan MI , Gupta D , Naru J , Singh SB , Mohanty AK , Vanamail P *et al*. (2016) Comparative proteomic analysis of advanced ovarian cancer tissue to identify potential biomarkers of responders and nonresponders to first‐line chemotherapy of carboplatin and paclitaxel. Biomark Cancer 8, 43–56. 26997873 10.4137/BIC.S35775 PMC4795487

[28] Zuehlke AD , Beebe K , Neckers L & Prince T (2015) Regulation and function of the human HSP90AA1 gene. Gene 570, 8–16. 26071189 10.1016/j.gene.2015.06.018 PMC4519370

[29] Zou M , Bhatia A , Dong H , Jayaprakash P , Guo J , Sahu D , Hou Y , Tsen F , Tong C , O'Brien K *et al*. (2017) Evolutionarily conserved dual lysine motif determines the non‐chaperone function of secreted Hsp90alpha in tumour progression. Oncogene 36, 2160–2171. 27721406 10.1038/onc.2016.375 PMC5386837

[30] Chu SH , Liu YW , Zhang L , Liu B , Li L , Shi JZ & Li L (2013) Regulation of survival and chemoresistance by HSP90AA1 in ovarian cancer SKOV3 cells. Mol Biol Rep 40, 1–6. 23135731 10.1007/s11033-012-1930-3

[31] Joshi S , Wang T , Araujo TLS , Sharma S , Brodsky JL & Chiosis G (2018) Adapting to stress ‐ chaperome networks in cancer. Nat Rev Cancer 18, 562–575. 29795326 10.1038/s41568-018-0020-9 PMC6108944

[32] Shee K , Wells JD , Ung M , Hampsch RA , Traphagen NA , Yang W , Liu SC , Zeldenrust MA , Wang L , Kalari KR *et al*. (2020) A transcriptionally definable subgroup of triple‐negative breast and ovarian cancer samples shows sensitivity to HSP90 inhibition. Clin Cancer Res 26, 159–170. 31558472 10.1158/1078-0432.CCR-18-2213 PMC6942625

[33] Liu H , Xiao F , Serebriiskii IG , O'Brien SW , Maglaty MA , Astsaturov I , Litwin S , Martin LP , Proia DA , Golemis EA *et al*. (2013) Network analysis identifies an HSP90‐central hub susceptible in ovarian cancer. Clin Cancer Res 19, 5053–5067. 23900136 10.1158/1078-0432.CCR-13-1115 PMC3778161

[34] Khandelwal A , Crowley VM & Blagg BSJ (2016) Natural product inspired N‐terminal Hsp90 inhibitors: from bench to bedside? Med Res Rev 36, 92–118. 26010985 10.1002/med.21351 PMC4659773

[35] Roe SM , Prodromou C , O'Brien R , Ladbury JE , Piper PW & Pearl LH (1999) Structural basis for inhibition of the Hsp90 molecular chaperone by the antitumor antibiotics radicicol and geldanamycin. J Med Chem 42, 260–266. 9925731 10.1021/jm980403y

[36] Tatokoro M , Koga F , Yoshida S & Kihara K (2015) Heat shock protein 90 targeting therapy: state of the art and future perspective. EXCLI J 14, 48–58. 26600741 10.17179/excli2014-586 PMC4652636

[37] Zhang Z , Xie Z , Sun G , Yang P , Li J , Yang H , Xiao S , Liu Y , Qiu H , Qin L *et al*. (2015) Reversing drug resistance of cisplatin by hsp90 inhibitors in human ovarian cancer cells. Int J Clin Exp Med 8, 6687–6701. 26221207 PMC4509152

[38] Ying W , Du Z , Sun L , Foley KP , Proia DA , Blackman RK , Zhou D , Inoue T , Tatsuta N , Sang J *et al*. (2012) Ganetespib, a unique triazolone‐containing Hsp90 inhibitor, exhibits potent antitumor activity and a superior safety profile for cancer therapy. Mol Cancer Ther 11, 475–484. 22144665 10.1158/1535-7163.MCT-11-0755

[39] Shimamura T , Perera SA , Foley KP , Sang J , Rodig SJ , Inoue T , Chen L , Li D , Carretero J , Li YC *et al*. (2012) Ganetespib (STA‐9090), a nongeldanamycin HSP90 inhibitor, has potent antitumor activity in *in vitro* and *in vivo* models of non‐small cell lung cancer. Clin Cancer Res 18, 4973–4985. 22806877 10.1158/1078-0432.CCR-11-2967 PMC3477583

[40] He S , Zhang C , Shafi AA , Sequeira M , Acquaviva J , Friedland JC , Sang J , Smith DL , Weigel NL , Wada Y *et al*. (2013) Potent activity of the Hsp90 inhibitor ganetespib in prostate cancer cells irrespective of androgen receptor status or variant receptor expression. Int J Oncol 42, 35–43. 23152004 10.3892/ijo.2012.1698 PMC3583620

[41] Lai CH , Park KS , Lee DH , Alberobello AT , Raffeld M , Pierobon M , Pin E , Petricoin Iii EF , Wang Y & Giaccone G (2014) HSP‐90 inhibitor ganetespib is synergistic with doxorubicin in small cell lung cancer. Oncogene 33, 4867–4876. 24166505 10.1038/onc.2013.439 PMC4002667

[42] Proia DA & Bates RC (2014) Ganetespib and HSP90: translating preclinical hypotheses into clinical promise. Cancer Res 74, 1294–1300. 24556722 10.1158/0008-5472.CAN-13-3263

[43] Kramer D , Stark N , Schulz‐Heddergott R , Erytch N , Edmunds S , Rossmann L , Bastians H , Concin N , Moll UM & Dobbelstein M (2017) Strong antitumor synergy between DNA crosslinking and HSP90 inhibition causes massive premitotic DNA fragmentation in ovarian cancer cells. Cell Death Differ 24, 300–316. 27834954 10.1038/cdd.2016.124 PMC5299713

[44] Eroglu Z , Chen YA , Gibney GT , Weber JS , Kudchadkar RR , Khushalani NI , Markowitz J , Brohl AS , Tetteh LF , Ramadan H *et al*. (2018) Combined BRAF and HSP90 inhibition in patients with unresectable BRAF (V600E)‐mutant melanoma. Clin Cancer Res 24, 5516–5524. 29674508 10.1158/1078-0432.CCR-18-0565 PMC6195480

[45] Piotrowska Z , Costa DB , Oxnard GR , Huberman M , Gainor JF , Lennes IT , Muzikansky A , Shaw AT , Azzoli CG , Heist RS *et al*. (2018) Activity of the Hsp90 inhibitor luminespib among non‐small‐cell lung cancers harboring EGFR exon 20 insertions. Ann Oncol 29, 2092–2097. 30351341 10.1093/annonc/mdy336

[46] Ray‐Coquard I , Braicu I , Berger R , Mahner S , Sehouli J , Pujade‐Lauraine E , Cassier PA , Moll UM , Ulmer H , Leunen K *et al*. (2019) Part I of GANNET53: a European multicenter phase I/II trial of the Hsp90 Inhibitor Ganetespib combined with weekly paclitaxel in women with high‐grade, platinum‐resistant epithelial ovarian cancer‐a study of the GANNET53 consortium. Front Oncol 9, 832. 31552170 10.3389/fonc.2019.00832 PMC6746955

[47] Concin N , Braicu I , Combe P , Laure Ray‐Coquard I , Joly F , Harter P , Wimberger P , Lotz J‐P , Ignatov A , Schmalfeldt B *et al*. (2018) Phase II results of GANNET53: A European multicenter phase I/randomized II trial of the Hsp90 inhibitor Ganetespib (G) combined with weekly Paclitaxel (P) in women with high‐grade serous, high‐grade endometrioid, or undifferentiated, platinum‐resistant epithelial ovarian, fallopian tube or primary peritoneal cancer. J Clin Oncol 36, 5567.

[48] Wang X , Song X , Zhuo W , Fu Y , Shi H , Liang Y , Tong M , Chang G & Luo Y (2009) The regulatory mechanism of Hsp90alpha secretion and its function in tumor malignancy. Proc Natl Acad Sci USA 106, 21288–21293. 19965370 10.1073/pnas.0908151106 PMC2795546

