# Supplementary material for: HSP90 identified by a proteomic approach as druggable target to reverse platinum resistance in ovarian cancer
Source: Mol Oncol. 2021 Jan 19;15(4):1005–23. doi: 10.1002/1878-0261.12883 (PMC8024727; doi:10.1002/1878-0261.12883)
Supplement: Supplementary file 1 — Fig. S1. Validation by Western blot of protein identified in the cellular models as differentially expressed in the 2‐D DIGE LC‐MS/MS analysis. Fig. S2. Ingenuity Pathway Analysis of all identified proteins. Fig. S3. mRNA expression in parental and Pt‐res EOC cell models. Fig. S4. Clonogenic assay of TOV‐112D and TOV‐112D Pt‐res cells. Fig. S5. Pro‐apoptotic effect of CDDP and/or ganetespib in Pt‐ MDAH Pt‐res and in TOV‐112D parental cells. Fig. S6. Effect of CDDP and/or ganetespib on HSP90α expression in TOV‐112D and TOV‐112D Pt‐res cells and on tumor growth of TOV‐112D parental cells xenograft model. [file MOL2-15-1005-s001.zip › mol212883-sup-0010-Figure Legends.docx]

**Supplementary Figure Legends**

**Supplementary Figure 1**. **Validation by Western blot of protein identified in the cellular**

**models as differentially expressed in the 2-D DIGE LC-MS/MS analysis**.

A 3D view of the 2D-DIGE quantification for each spot is illustrated. The numbers indicate the fold change values in spot levels from 2-D DIGE data relative to parental cells as reported in Supplementary Table 1, 2 and 3. β-actin and GAPDH were used as loading controls. Western blot quantification was performed by ImageJ software.

**Supplementary Figure 2**. I**ngenuity Pathway Analysis of all identified proteins.**

**A**, **B**, **C**, Visual representation of the network generated by IPA software including the proteins differentially expressed (colored in orange) in TOV-112D, MDAH and OVSAHO cells respectively (direct interactions), with HSP90 emerging as the main hub (colored in green). D, IPA network of all differentially expressed proteins identified in the three cellular models (colored in orange) (direct interaction), with HSP90 maintaining a central hub role (colored in green).

**Supplementary Figure 3**. **mRNA expression in parental and Pt-res EOC cell models.**

Graphs reporting mRNA expression of HSP90α and HSP90β in parental and Pt-res cells determined by qRT-PCR. mRNA levels were analyzed in triplicate and normalized to GAPDH housekeeping gene expression.

**Supplementary Figure 4**. **Clonogenic assay of TOV-112D and TOV-112D Pt-res cells.**

Clonogenic assay of TOV-112D (**A**) and TOV-112D Pt-res cells (**B-C**) treated with CDDP, ganetespib, or their combination (simultaneous or sequential exposure with 24 hours delay to either drug) at the IC10 72h doses for parental cells. Representative data of at least three independent experiments performed in triplicates. Statistically significant results are reported (a indicates control group, b indicates CDDP-treated cells and c indicates ganetespib-treated cells *p<0.05, **p<0.01, ***p<0.001 and ****p<0.0001, ns not statistically significant).

**Supplementary Figure 5**. **Pro-apoptotic effect of CDDP and/or ganetespib in Pt- MDAH Pt-res and in TOV-112D parental cells.**

**A**, Western blot analysis of γH2AX, PARP1 and caspase 3 cleavage in MDAH Pt-res pool 2 cells untreated or treated with CDDP and/or ganetespib at IC50 72h doses for parental cells. β-actin expression serves as loading control. **B**, Apoptotis and necrosis evaluated by flow cytometry after Annexin V-FITC and Propidium Iodide staining in TOV-112D parental cells, untreated or treated for 48 hours (upper panels) or 72 hours (lower panels), with CDDP and/or ganetespib at IC50 72h doses. **C**, Western blot analysis of PARP1 cleavage in TOV-112D parental cells untreated or treated with CDDP and/or ganetespib at IC50 72h doses. β-actin expression serves as loading control. Western blot quantification was performed by ImageJ software.

**Supplementary Figure 6. Effect of CDDP and/or ganetespib on HSP90α expression in TOV-112D and TOV-112D Pt-res cells and on tumor growth of TOV-112D parental cells** **xenograft model.**

**A**, Western blot analysis of HSP90α in TOV-112D and TOV-112D Pt-res cells untreated or treated with CDDP and/or ganetspib at IC50 72h doses for parental cells. β-actin expression serves as loading control. Western blot quantification was performed by ImageJ software.

**B-C**, Graph reporting the subcutaneous growth of TOV-112D parental cells in both flanks of nude mice treated with vehicles (UNT), carboplatin (CBDCA) (15 mg/Kg dissolved in PBS), ganetespib (GANE) (30mg/Kg dissolved in 10% DMSO+ 40% PEG+ 50% ddH2O), or combination of both, three times/week for one week. Results are expressed as fold increase of tumor volume (respect to the day 8 from injection) in B and as scattered dot plot (n=8) with median (black bar) of tumor volume in C.
