# Supplementary figures and images for: HSP90 identified by a proteomic approach as druggable target to reverse platinum resistance in ovarian cancer
Source: Mol Oncol. 2021 Jan 19;15(4):1005–23. doi: 10.1002/1878-0261.12883 (PMC8024727; doi:10.1002/1878-0261.12883)

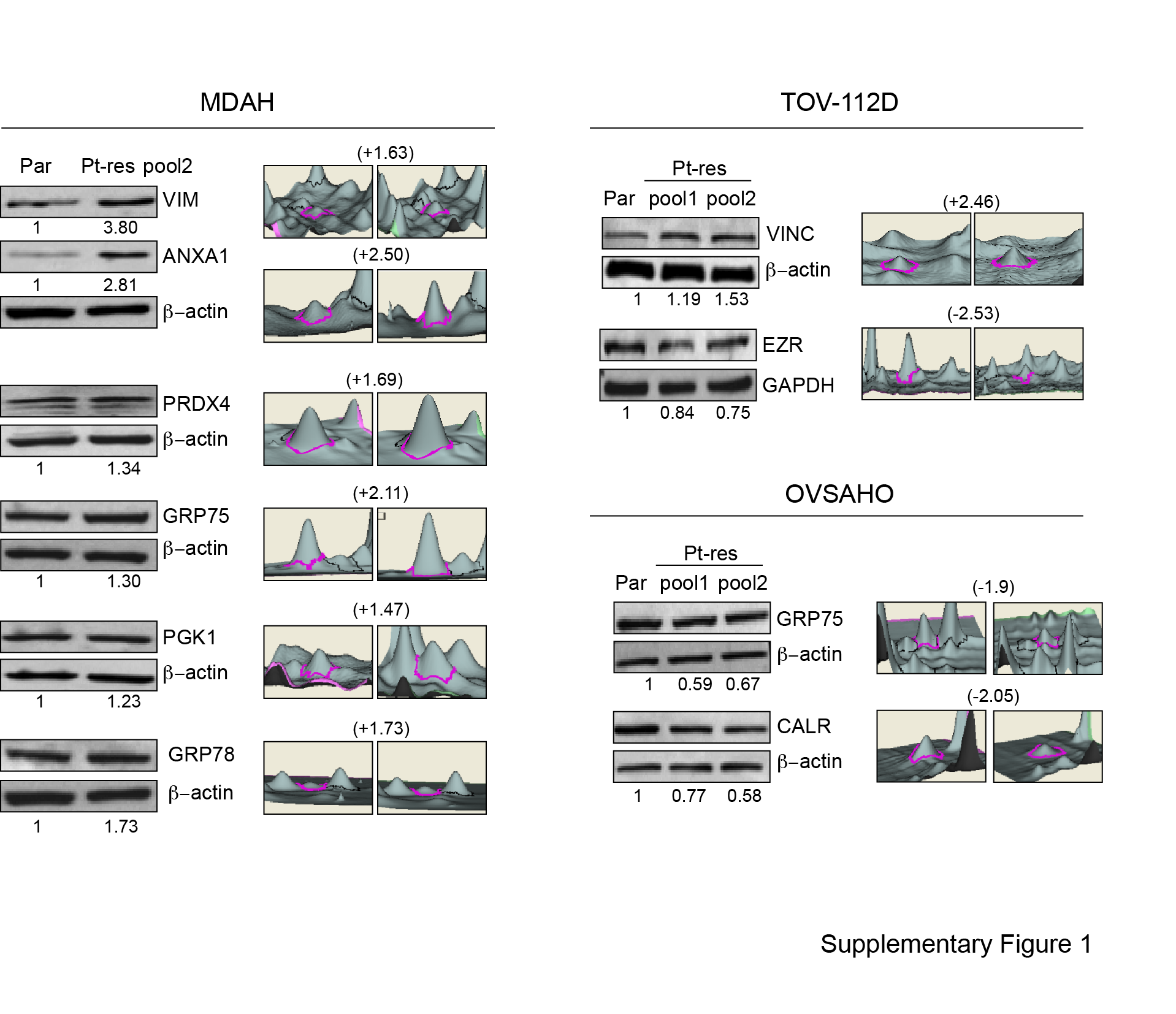

Supplement: Supplementary file 1 — Fig. S1. Validation by Western blot of protein identified in the cellular models as differentially expressed in the 2‐D DIGE LC‐MS/MS analysis. Fig. S2. Ingenuity Pathway Analysis of all identified proteins. Fig. S3. mRNA expression in parental and Pt‐res EOC cell models. Fig. S4. Clonogenic assay of TOV‐112D and TOV‐112D Pt‐res cells. Fig. S5. Pro‐apoptotic effect of CDDP and/or ganetespib in Pt‐ MDAH Pt‐res and in TOV‐112D parental cells. Fig. S6. Effect of CDDP and/or ganetespib on HSP90α expression in TOV‐112D and TOV‐112D Pt‐res cells and on tumor growth of TOV‐112D parental cells xenograft model. [file MOL2-15-1005-s001.zip › mol212883-sup-0001-FigS1.png]

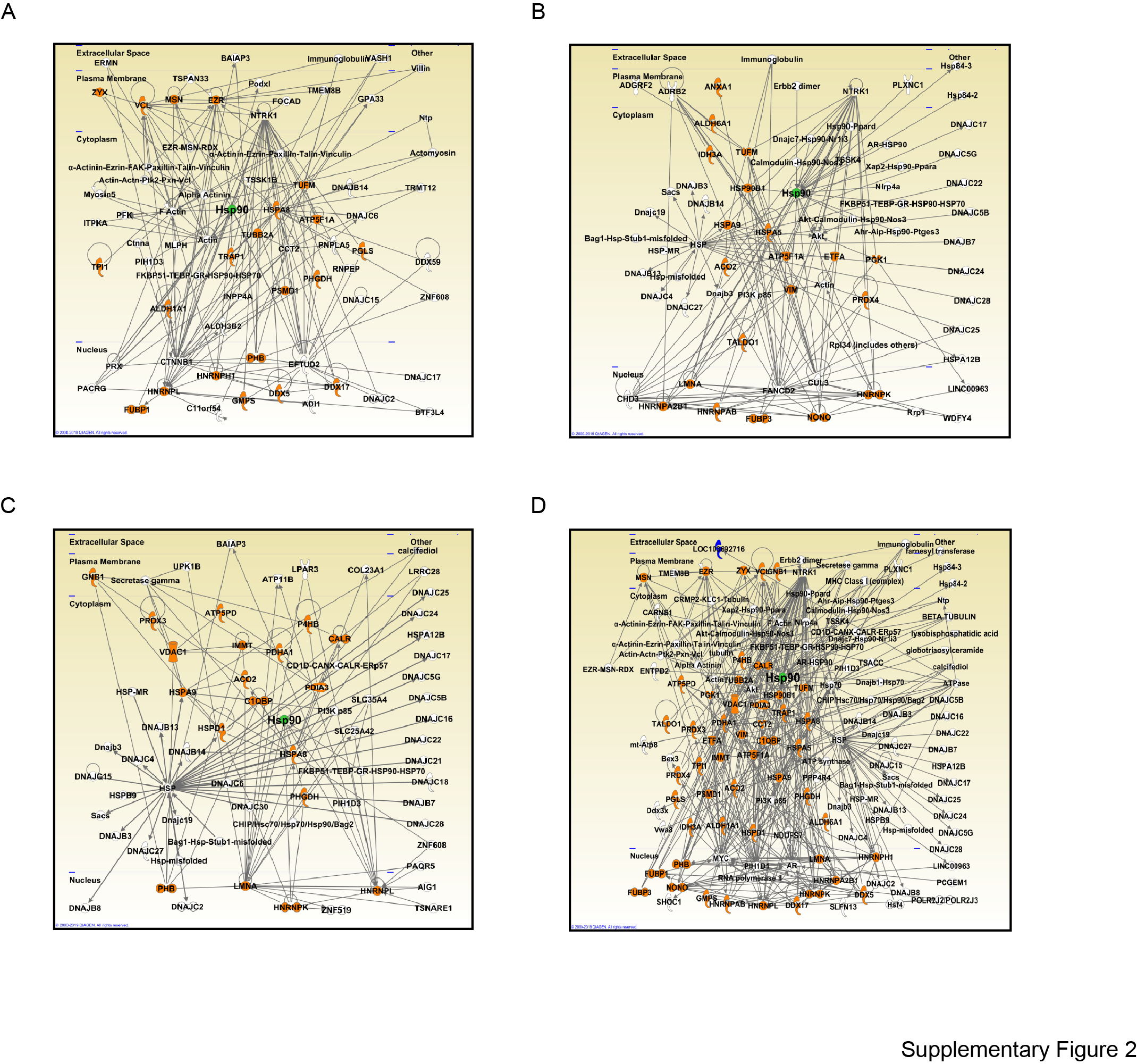

Supplement: Supplementary file 1 — Fig. S1. Validation by Western blot of protein identified in the cellular models as differentially expressed in the 2‐D DIGE LC‐MS/MS analysis. Fig. S2. Ingenuity Pathway Analysis of all identified proteins. Fig. S3. mRNA expression in parental and Pt‐res EOC cell models. Fig. S4. Clonogenic assay of TOV‐112D and TOV‐112D Pt‐res cells. Fig. S5. Pro‐apoptotic effect of CDDP and/or ganetespib in Pt‐ MDAH Pt‐res and in TOV‐112D parental cells. Fig. S6. Effect of CDDP and/or ganetespib on HSP90α expression in TOV‐112D and TOV‐112D Pt‐res cells and on tumor growth of TOV‐112D parental cells xenograft model. [file MOL2-15-1005-s001.zip › mol212883-sup-0002-FigS2.png]

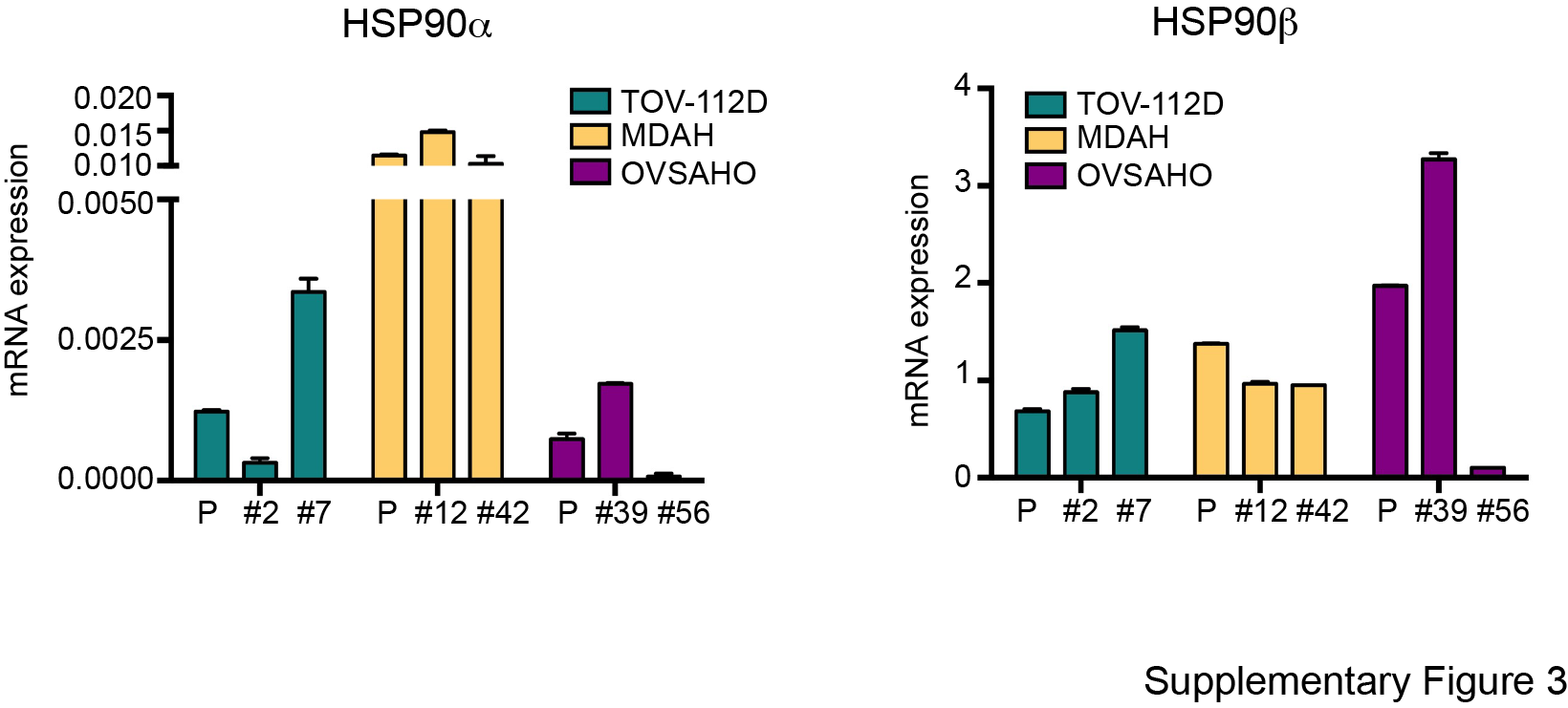

Supplement: Supplementary file 1 — Fig. S1. Validation by Western blot of protein identified in the cellular models as differentially expressed in the 2‐D DIGE LC‐MS/MS analysis. Fig. S2. Ingenuity Pathway Analysis of all identified proteins. Fig. S3. mRNA expression in parental and Pt‐res EOC cell models. Fig. S4. Clonogenic assay of TOV‐112D and TOV‐112D Pt‐res cells. Fig. S5. Pro‐apoptotic effect of CDDP and/or ganetespib in Pt‐ MDAH Pt‐res and in TOV‐112D parental cells. Fig. S6. Effect of CDDP and/or ganetespib on HSP90α expression in TOV‐112D and TOV‐112D Pt‐res cells and on tumor growth of TOV‐112D parental cells xenograft model. [file MOL2-15-1005-s001.zip › mol212883-sup-0003-FigS3.png]

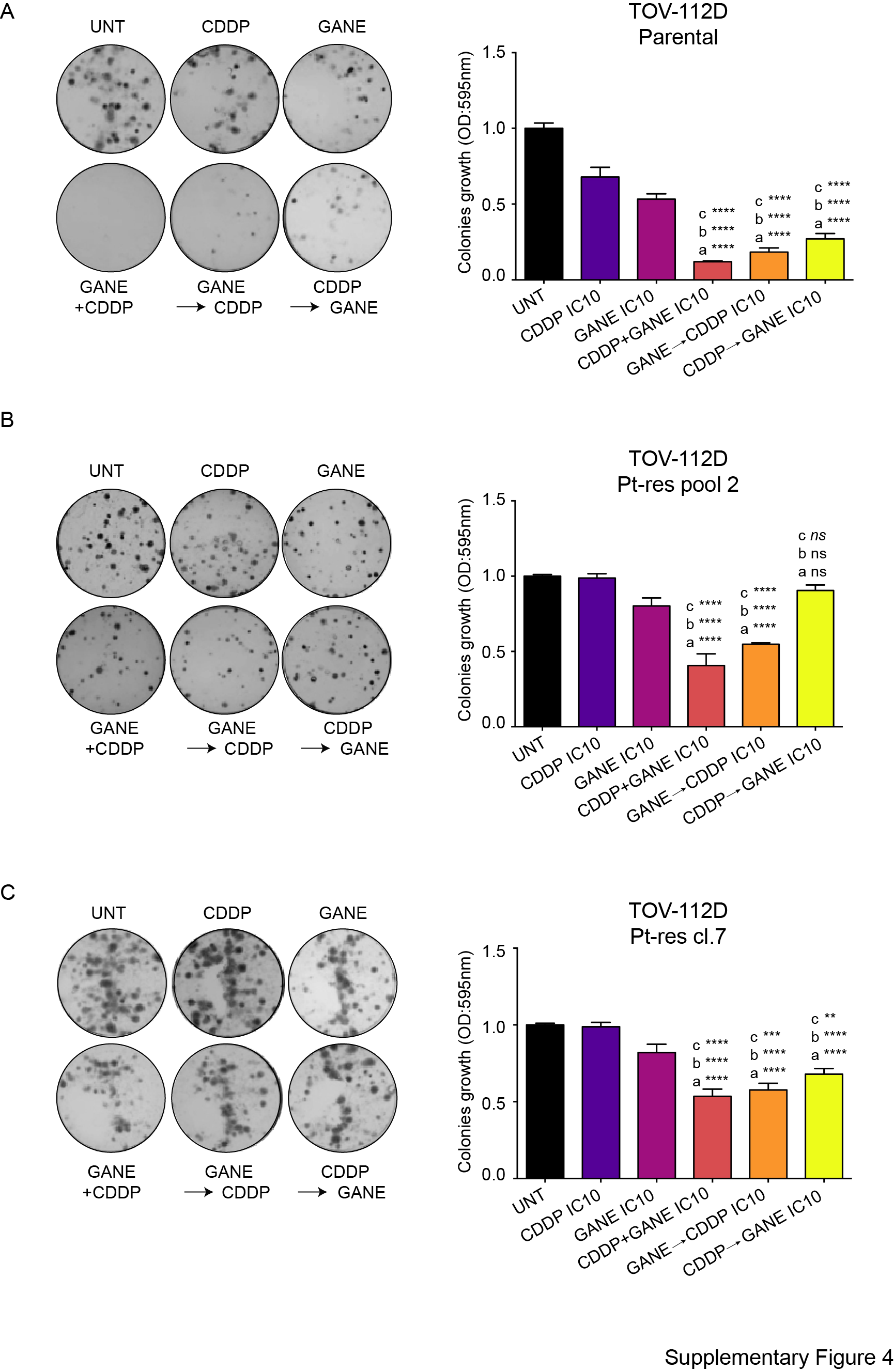

Supplement: Supplementary file 1 — Fig. S1. Validation by Western blot of protein identified in the cellular models as differentially expressed in the 2‐D DIGE LC‐MS/MS analysis. Fig. S2. Ingenuity Pathway Analysis of all identified proteins. Fig. S3. mRNA expression in parental and Pt‐res EOC cell models. Fig. S4. Clonogenic assay of TOV‐112D and TOV‐112D Pt‐res cells. Fig. S5. Pro‐apoptotic effect of CDDP and/or ganetespib in Pt‐ MDAH Pt‐res and in TOV‐112D parental cells. Fig. S6. Effect of CDDP and/or ganetespib on HSP90α expression in TOV‐112D and TOV‐112D Pt‐res cells and on tumor growth of TOV‐112D parental cells xenograft model. [file MOL2-15-1005-s001.zip › mol212883-sup-0004-FigS4.png]

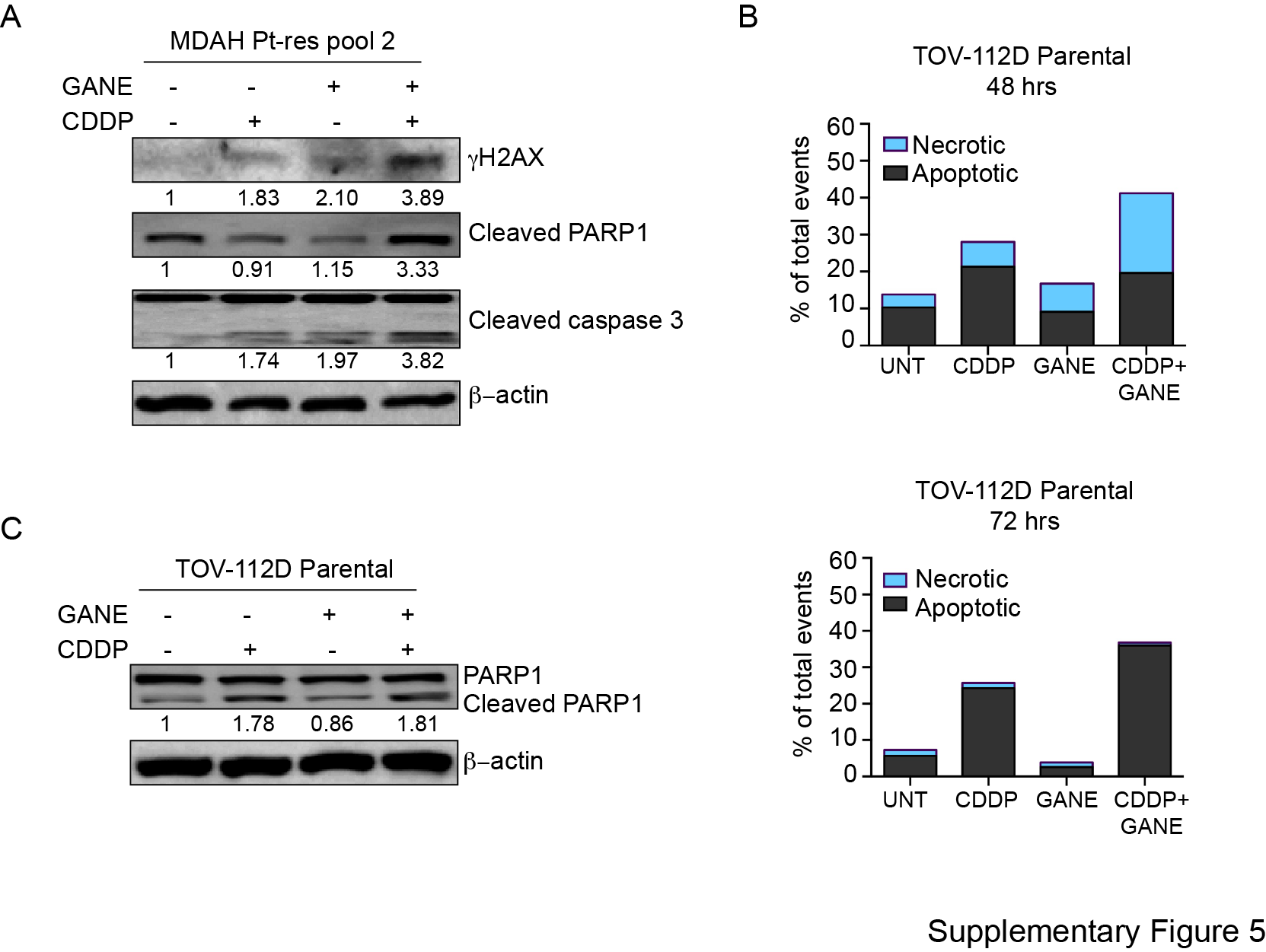

Supplement: Supplementary file 1 — Fig. S1. Validation by Western blot of protein identified in the cellular models as differentially expressed in the 2‐D DIGE LC‐MS/MS analysis. Fig. S2. Ingenuity Pathway Analysis of all identified proteins. Fig. S3. mRNA expression in parental and Pt‐res EOC cell models. Fig. S4. Clonogenic assay of TOV‐112D and TOV‐112D Pt‐res cells. Fig. S5. Pro‐apoptotic effect of CDDP and/or ganetespib in Pt‐ MDAH Pt‐res and in TOV‐112D parental cells. Fig. S6. Effect of CDDP and/or ganetespib on HSP90α expression in TOV‐112D and TOV‐112D Pt‐res cells and on tumor growth of TOV‐112D parental cells xenograft model. [file MOL2-15-1005-s001.zip › mol212883-sup-0005-FigS5.png]

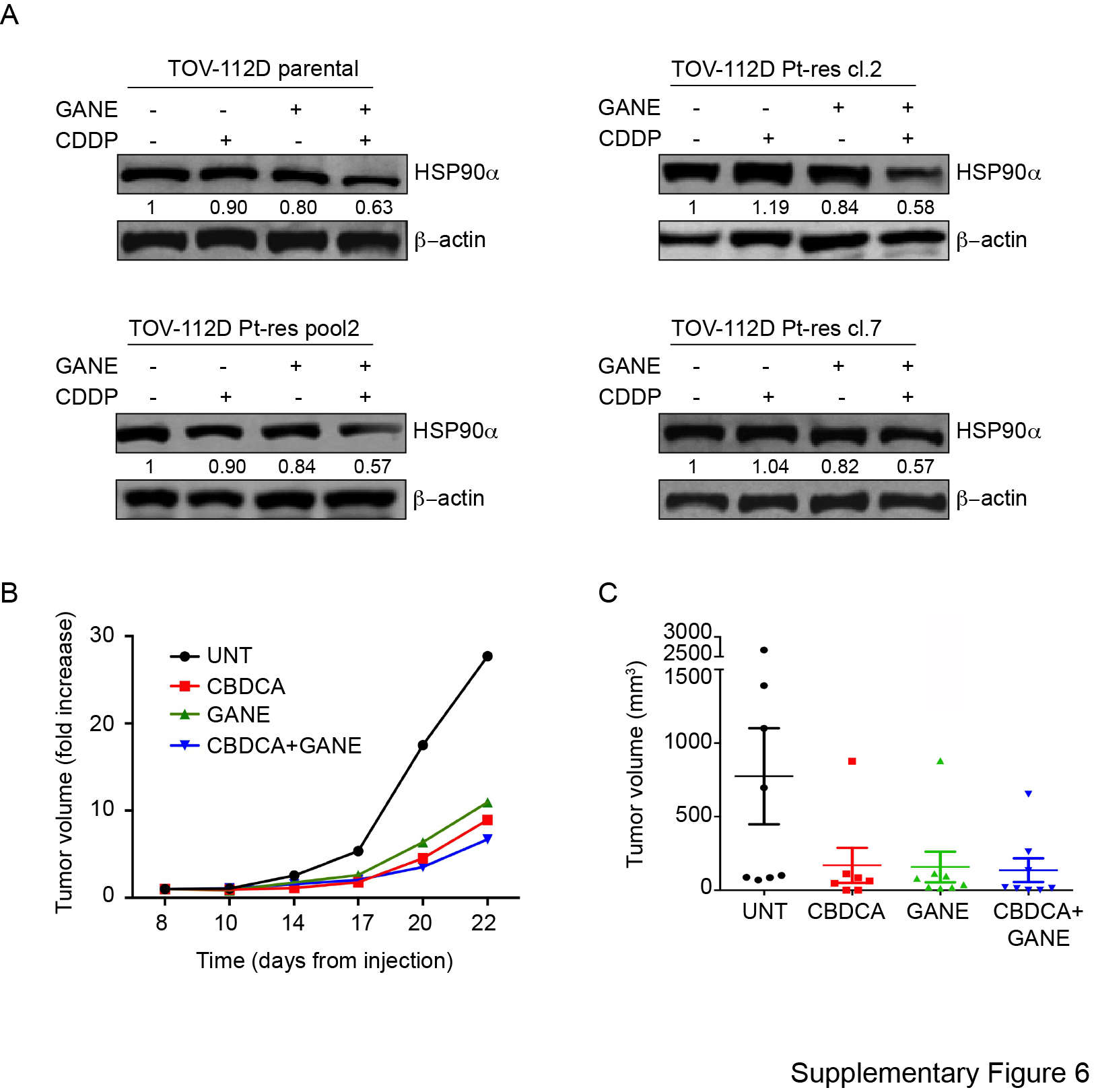

Supplement: Supplementary file 1 — Fig. S1. Validation by Western blot of protein identified in the cellular models as differentially expressed in the 2‐D DIGE LC‐MS/MS analysis. Fig. S2. Ingenuity Pathway Analysis of all identified proteins. Fig. S3. mRNA expression in parental and Pt‐res EOC cell models. Fig. S4. Clonogenic assay of TOV‐112D and TOV‐112D Pt‐res cells. Fig. S5. Pro‐apoptotic effect of CDDP and/or ganetespib in Pt‐ MDAH Pt‐res and in TOV‐112D parental cells. Fig. S6. Effect of CDDP and/or ganetespib on HSP90α expression in TOV‐112D and TOV‐112D Pt‐res cells and on tumor growth of TOV‐112D parental cells xenograft model. [file MOL2-15-1005-s001.zip › mol212883-sup-0006-FigS6.png]
