# Supplementary material for: HSP90 identified by a proteomic approach as druggable target to reverse platinum resistance in ovarian cancer
Source: Mol Oncol. 2021 Jan 19;15(4):1005–23. doi: 10.1002/1878-0261.12883 (PMC8024727; doi:10.1002/1878-0261.12883)
Supplement: Supplementary file 2 — Table S1. Differentially expressed proteins identified by mass spectrometry in TOV‐112D model. Table S2. Differentially expressed proteins identified by mass spectrometry in MDAH model. Table S3. Differentially expressed proteins identified by mass spectrometry in OVSAHO model. [file MOL2-15-1005-s002.docx]

| **Spot no ^a)^** | **Spot name ^b)^** | **Description** | **AC ^c)^** | **Theoretical pI/Mr (kDa)** | **Mascot search results** | | | |
| --- | --- | --- | --- | --- | --- | --- | --- | --- |
|  |  |  |  |  | **No. of peptides (unique peptides)** | **Sequence coverage (%)** | **Score** | **Fold change ^d)^**  **TOV-112D Pt-res pool/ TOV-112D** |
| **EMT, cancer stem cells, cytoskeleton** | | | | | | | | |
| 191 | VINC_HUMAN | Vinculin | P18206 | 5.5/124292 | 3(3) | 3 | 35 | **+2.46** |
| 485 | ZYX_HUMAN | Zyxin | Q15942 | 6.22/62436 | 2(1) | 2 | 42 | **-1.7** |
| 509 | EZRI_HUMAN | Ezrin | P15311 | 5.94/69484 | 43(21) | 32 | 286 | **-2.53** |
| 513 | EZRI_HUMAN | Ezrin PPP | P15311 | 5.94/69484 | 6(6) | 8 | 34 | **-2.15** |
| 693 | MSN_HUMAN | Moesin | P26038 | 6.08/67890 | 4(4) | 7 | 97 | **-1.87** |
| 1133 | ALDH1A1_HUMAN | Retinaldehydrogenase 1 | P00352 | 6.3/55454 | 50(22) | 54 | 561 | **-1.72** |
| 1536 | TBB2A_HUMAN | Tubulin beta-2A chain | Q13885 | 4.78/50274 | 18(10) | 27 | 371 | **-6.78** |
| **Stress response, chaperones and protein fate** | | | | | | | | |
| 617 | TRAP1_HUMAN | Heat shock protein 75 kDa | Q12931 | 8.3/80345 | 63(23) | 35 | 756 | **-1.79** |
| 681 | HSP7C_HUMAN | Heat shock cognate 71 kDa | P11142 | 5.37/71082 | 50(15) | 26 | 782 | **-2.08** |
| 1155 | ATPA_HUMAN | ATP synthase subunit alpha | P25705 | 9.10/59828 | 3(3) | 6 | 50 | **-2.38** |
| 1119 | TCPB_HUMAN | T-complex protein 1 subunit beta | P78371 | 6.01/57794 | 302(24) | 59 | 6970 | **-1.67** |
| 2247 | EFTU_HUMAN | Elongation factor Tu | P49411 | 7.26/49852 | 18(11) | 28 | 311 | **+1.95** |
| **Metabolism** | | | | | | | | |
| 680 | GMPS_HUMAN | GMP synthase [glutamine-hydrolyzing] | P49915 | 6.42/77408 | 16(11) | 16 | 196 | **-1.8** |
| 1134 | SERA_HUMAN  (3- PHGDH) | D-3-phosphoglycerate dehydrogenase | O43175 | 6.29/57356 | 51(13) | 28 | 881 | **-1.71** |
| 2574 | PGLS_HUMAN | 6-phosphogluconolactonase | O95336 | 5.7/27815 | 7(4) | 19 | 83 | **+1.85** |
| 2744 | TPI1_HUMAN | Triose phosphate isomerase OS | P60174 | 5.65/31057 | 8(5) | 20 | 168 | **+2.47** |
| **Nucleic acid processing and DNA damage** | | | | | | | | |
| 192 | PSMD1_HUMAN | 26S proteasome non-ATPase regulatory  subunit 1 | Q99460 | 5.25/106795 | 3(3) | 3 | 39 | **+1.72** |
| 663 | DDX17_HUMAN | Probable ATP-dependent RNA helicase DDX17 | Q92841 | 8.53/80906 | 10(10) | 15 | 262 | **-1.66** |
| 668 | FUBP1_HUMAN | Far upstream element-binding  protein 1 | Q92945 | 7.18/67690 | 13(11) | 18 | 147 | **-1.5** |
| 783 | DDX5_HUMAN | Probable ATP-dependent RNA helicase DDX5 | P17844 | 9.06/69618 | 3(3) | 5 | 76 | **-2.01** |
| 857 | HNRPL_HUMAN | Heterogeneousnuclear  ribonucleoprotein L | P14866 | 8.46/64720 | 8(7) | 13 | 192 | **-2.42** |
| 2396 | PHB_HUMAN | Prohibitin | P35232 | 5.57/29843 | 17(9) | 38 | 306 | **+1.83** |
| 2813 | HNRH1_HUMAN | Heterogeneousnuclear  ribonucleoprotein H | P31943 | 5.89/49484 | 8(3) | 8 | 224 | **+1.57** |

**Supplementary Table 1. Differentially expressed proteins identified by mass spectrometry in TOV-112D model.**

^a)^ Master spot numbers

^b)^Protein acrostic names according UniProtKB

^c)^UniProtKB Accession Numbers

^d)^Average ratio between the TOV-112D Pt-res pool and TOV-112D cells are reported and significant values indicated (≤ 1.3 or ≥ 1.3)

In grey, proteins shared in at least two cellular models.

**Supplementary Table 2. Differentially expressed proteins identified by mass spectrometry in MDAH model.**

| **Spot no ^a)^** | **Spot name ^b)^** | **Description** | **AC ^c)^** | **Theoretical pI/Mr (kDa)** | **Mascot search results** | | |  |
| --- | --- | --- | --- | --- | --- | --- | --- | --- |
|  |  |  |  |  | **No. of peptides (unique peptides)** | **Sequence coverage (%)** | **Score** | **Fold change ^d)^**  **MDAH Pt-res pool/ MDAH** |
| **EMT, cancer stem cells, cytoskeleton** | | | | | | | | |
| 433 | ALDH6A1_HUMAN | Methylmalonate-semialdehydedehydrogenase | Q02252 | 8.72/58259 | 6(6) | 11 | 146 | **+1.59** |
| 479 | VIME_HUMAN | Vimentin | P08670 | 5.06/53676 | 16(13) | 27 | 463 | **+1.63** |
| 1136 | ANXA1_HUMAN | Annexin A1 | P04083 | 6.57/38918 | 15(10) | 37 | 570 | **+2.50** |
| **Stress response, chaperones and protein fate** | | | | | | | | |
| 287 | GRP78_HUMAN | 78 kDaglucose-regulatedprotein | P11021 | 5.07/72402 | 238(26) | 49 | 6110 | **+1.73** |
| 292 | GRP75_HUMAN | Stress-70 protein | P38646 | 5.87/73920 | 125(34) | 40 | 1714 | **+2.11** |
| 498 | ENPL_HUMAN | Endoplasmin | P14625 | 4.76/92696 | 36(15) | 79 | 1023 | **+1.58** |
| 591 | ATPA_HUMAN | ATP synthase subunit alpha, mitochondrial | P25705 | 9.16/59828 | 8(7) | 15 | 262 | **-1.83** |
| 788 | EFTU_HUMAN | Elongation factor Tu | P49411 | 7.26/49852 | 6(5) | 12 | 225 | **+3.41** |
| **Metabolism** | | | | | | | | |
| 855 | PGK1_HUMAN | Phosphoglyceratekinase 1 | P00558 | 8.3/44985 | 13(11) | 30 | 369 | **+1.47** |
| 985 | IDH3A_HUMAN | Isocitratedehydrogenase [NAD] subunit alpha | P50213 | 6.47/40022 | 10(7) | 21 | 302 | **+2.25** |
| 987 | TALDO_HUMAN | Transaldolase | P37837 | 6.36/37688 | 2(2) | 7 | 72 | **+1.45** |
| 1233 | ETFA_HUMAN | Electron transfer flavoprotein subunit alpha | P13804 | 8.62/35400 | 2(2) | 9 | 117 | **+1.37** |
| **Nucleic acid processing and DNA damage** | | | | | | | | |
| 166 | ACO2_HUMAN | Aconitatehydratase, mitochondrial | Q99798 | 7.36/86113 | 21(16) | 22 | 367 | **+1.47** |
| 432 | FUBP3_HUMAN | Far upstream element-binding protein 3 | Q96I24 | 8.60/61944 | 8(8) | 13 | 102 | **+1.43** |
| 504 | NONO_HUMAN | Non-POU domain-containing octamer-binding protein | Q15233 | 9.01/54311 | 17(13) | 19 | 107 | **-1.50** |
| 582 | HNRPK_HUMAN | Heterogeneous nuclear ribonucleoprotein K | P61978 | 5.39/51230 | 13(10) | 28 | 496 | **+1.62** |
| 931 | ROAA_HUMAN | Heterogeneous nuclear ribonucleoprotein A/B | Q99729 | 8.22/36313 | 6(4) | 12 | 146 | **+2.44** |
| 1216 | ROA2_HUMAN | Heterogeneous nuclear ribonucleoproteins A2/B1 | P22626 | 8.97/37464 | 3(3) | 8 | 77 | **+2.09** |
| 1416 | PRDX4_HUMAN | Peroxiredoxin-4 | Q13162 | 5.86/30749 | 5(5) | 19 | 119 | **+1.69** |
| **Nuclear Lamina** | | | | | | | | |
| 315 | LMNA_HUMAN | Prelamin-A/C | P02545 | 6.57/74380 | 22(21) | 34 | 667 | **-1.61** |

^a)^ Master spot numbers

^b)^Protein acrostic names according UniProtKB

^c)^UniProtKB Accession Numbers

^d)^Average ratio between the MDAH Pt-res pool and MDAH cells are reported and significant values indicated (≤ 1.3 or ≥ 1.3)

In grey, proteins shared in at least two cellular models.

**Supplementary Table 3. Differentially expressed proteins identified by mass spectrometry in OVSAHO model.**

| **Spot no ^a)^** | **Spot name^b)^** | **Description** | **AC ^c)^** | **Theoretical pI/Mr (kDa)** | **Mascot search results** | | |  |
| --- | --- | --- | --- | --- | --- | --- | --- | --- |
|  |  |  |  |  | **No. of peptides (unique peptides)** | **Sequence coverage (%)** | **Score** | **Fold change ^d)^**  **OVSAHO Pt-res pool/ OVSAHO** |
| **Stress response, chaperones and protein fate** | | | | | | | | |
| 505 | GRP75_HUMAN | Stress-70 protein, | P38646 | 5.87/73920 | 227(30) | 50 | 5079 | **-1.9** |
| 531 | HSP7C_HUMAN | Heat shock cognate 71 kDa | P11142 | 5.37/71082 | 67(17) | 28 | 1504 | **-1.73** |
| 794 | CH60_HUMAN | 60 kDa heat shock protein | P10809 | 5.7/61187 | 340(24) | 56 | 8123 | **+1.38** |
| 822 | PDIA1_HUMAN | Protein disulfide-isomerase 1 **PPP** | P07237 | 4.76/57480 | 6(6) | 12 | 70 | **-2.2** |
| 838 | PDIA1_HUMAN | Protein disulfide-isomerase 1 | P07237 | 4.76/57480 | 81(21) | 43 | 775 | **-1.62** |
| 886 | PDIA3_HUMAN | Protein disulfide-isomerase A3 **P** | P30101 | 5.98/57146 | 9(9) | 19 | 237 | **-1.89** |
| 887 | PDIA3_HUMAN | Protein disulfide-isomerase A3 | P30101 | 5.98/57146 | 85(16) | 35 | 1064 | **-1.9** |
| 2110 | GBB1_HUMAN | Guanine nucleotide-binding protein subunit beta-1 | P62873 | 5.6/38151 | 3(3) | 9 | 72 | **+1.42** |
| 2941 | ATP5H_HUMAN | ATP synthase subunit d | O75947 | 5.21/18537 | 16(5) | 34 | 249 | **-1.97** |
| **Metabolism** | | | | | | | | |
| 292 | IMMT_HUMAN | Mitochondrial inner membrane protein **PPP** | Q16891 | 6.08/84026 | 29(20) | 29 | 660 | **-1.62** |
| 320 | IMMT_HUMAN | Mitochondrial  inner membrane protein **P** | Q16891 | 6.08/84026 | 41(24) | 39 | 680 | **-1.65** |
| 323 | IMMT_HUMAN | Mitochondrial inner membrane protein | Q16891 | 6.08/84026 | 18(13) | 17 | 264 | **-1.61** |
| 927 | SERA_HUMAN ([PHGDH](https://www.genenames.org/data/gene-symbol-report/#!/hgnc_id/HGNC:8923)**)** | D-3-phosphoglycerate dehydrogenase | O43175 | 6.29/57356 | 8(6) | 15 | 148 | **-1.66** |
| 1312 | ODPA_HUMAN | Pyruvate dehydrogenase E1 component subunit alpha | P08559 | 8.35/43952 | 4(4) | 10 | 87 | **-1.73** |
| 1986 | VDAC1_HUMAN | Voltage-dependent anion-selective channel protein 1 | P21796 | 8.62/38868 | 6(6) | 29 | 176 | **-1.94** |
| **Nucleic acid processing and DNA damage** | | | | | | | | |
| 315 | ACO2_HUMAN | Aconitate hydratase | Q99798 | 7.36/86113 | 11(9) | 13 | 116 | **-1.54** |
| 723 | HNRPL_HUMAN | Heterogeneous nuclear ribonucleoprotein L | P14866 | 8.46/64720 | 12(10) | 21 | 166 | **-1.43** |
| 751 | HNRPK_HUMAN | Heterogeneous nuclear ribonucleoprotein K | P61978 | 5.39/51230 | 55(16) | 41 | 683 | **+2.18** |
| 2091 | C1QBP_HUMAN | Complement component 1 Q binding protein | Q07021 | 4.74/31742 | 3(3) | 12 | 111 | **+2.32** |
| 2232 | PHB_HUMAN | Prohibitin | P35232 | 5.57/29843 | 10(6) | 29 | 242 | **+1.71** |
| 2685 | PRDX3_HUMAN | Thioredoxin-dependent peroxide reductase, | P30048 | 7.67/28017 | 6(4) | 16 | 136 | **-1.63** |
| **Nuclear Lamina** | | | | | | | | |
| 928 | LMNA_HUMAN | Prelamin-A/C | P02545 | 6.57/74380 | 6(6) | 9 | 112 | **-2.2** |
| **Immune response** | | | | | | | | |
| 1301 | CALR_HUMAN | Calreticulin | P27797 | 4.29/48283 | 88(11) | 29 | 2241 | **-2.05** |

^a)^ Master spot numbers

^b)^Protein acrostic names according UniProtKB

^c)^UniProtKB Accession Numbers

^d)^Average ratio between the OVSAHO Pt-res pool and OVSAHO cells are reported and significant values indicated (≤ 1.3 or ≥ 1.3)

In grey, proteins shared in at least two cellular models.
