# Supplementary material for: HSP90 identified by a proteomic approach as druggable target to reverse platinum resistance in ovarian cancer
Source: Mol Oncol. 2021 Jan 19;15(4):1005–23. doi: 10.1002/1878-0261.12883 (PMC8024727; doi:10.1002/1878-0261.12883)
Supplement: Supplementary file 3 — Table S4. Sensitivity of EOC cell lines to single agent treatments. [file MOL2-15-1005-s003.docx]

**Supplementary Table 4.**  **Sensitivity of EOC cell lines to single agent treatment**

| **CELL LINES** | **CDDP**  **IC_50_ (µM)**  **72h±SD** | **17AAG**  **IC_50_ (nM)**  **72h±SD** | **GANE**  **IC_50_ (nM)**  **72h±SD** | **DOXORUBICIN**  **IC_50_ (nM)**  **72h±SD** | **CDDP**  **IC_50_ (µM)**  **96h±SD** | **GANE**  **IC_50_ (nM)**  **96h±SD** |
| --- | --- | --- | --- | --- | --- | --- |
| **TOV-112D** | 4.45±0.57 | 105.50±29.22 | 13.87±1.80 | 19.76±3.19 | 2.25±0.09 | 13.10±2.15 |
| **TOV-112D**  **Pt-res pool 1** | 14.88± 3.09 | 410.82±3.26 | 25.46±0.96 | n.d. | n.d. | n.d. |
| **TOV-112D**  **Pt-res pool 2** | 10.20±1.47 | 191.06±17.20 | 23.20±2.36 | n.d | 4.73±0.32 | 20.15±0.61 |
| **TOV-112D**  **Pt-res cl. #2** | n.d. | n.d. | n.d. | 45.50±3.81 | 7.30±0.62 | 20.70±2.96 |
| **TOV-112D**  **Pt-res cl. #7** | n.d. | n.d. | n.d. | 88.20±13.43 | 7.62±0.30 | 24.09±2.24 |
| **MDAH** | 4.15±0.70 | 71.14±7.85 | 16.15±2.22 | n.d. | 2.45±0.77 | 19.65±4.45 |
| **MDAH**  **Pt-res pool 2** | 12.61±1.12 | 260.33±34.16 | 16.71±1.44 | n.d. | 7.90±0.98 | 14.30±0.42 |
| **MDAH**  **Pt-res cl. #42** | 8.30±0.42 | 175.09±0.01 | 18.90±3.81 | n.d. | 5.46±0.15 | 14.25±0.49 |
| **MDAH**  **Pt-res cl. #12** | 7.62±0.53 | 74.85±20.00 | 17.03±1.85 | n.d. | 5.76±0.20 | 17.30±2.86 |

Abbreviations: CDDP: Cisplatin; GANE: ganetespib; 17AAG: Tanespimycin.

IC: Inhibitory Concentration. The IC_50_ values were computed at 72h or 96h of treatment (mean±SD) from at least three separate experiments performed in triplicates. Cell growth assessment was done by sulforhodamine B colorimetric assay (see Materials and Methods).
