# Supplementary material for: HSP90 identified by a proteomic approach as druggable target to reverse platinum resistance in ovarian cancer
Source: Mol Oncol. 2021 Jan 19;15(4):1005–23. doi: 10.1002/1878-0261.12883 (PMC8024727; doi:10.1002/1878-0261.12883)
Supplement: Supplementary file 4 — Table S5. Combination index (CI) and dose reduction index (DRI) values for 17AAG and CDDP combination treatment accordingly to different cytotoxic ratio. Table S6. Combination index (CI) and dose reduction index (DRI) values for ganetespib and CDDP combination treatment accordingly to different cytotoxic ratio. Table S7. Combination index (CI) and dose reduction index (DRI) values for ganetespib and doxorubicin combination treatment at equitoxic ratio. [file MOL2-15-1005-s004.docx]

**Supplementary Table 5. Combination index (CI) and dose reduction index (DRI) values for**

**17AAG and CDDP combination treatment accordingly to different cytotoxic ratio**

|  | **17AAG-CDDP 50:50** | | | | | **17AAG-CDDP 75:25** | | | | | **17AAG-CDDP 25:75** | | | | |
| --- | --- | --- | --- | --- | --- | --- | --- | --- | --- | --- | --- | --- | --- | --- | --- |
| **CELL LINES** | **CI50±**  **SD** | **CI75±**  **SD** | **CI90±**  **SD** | **DRI50±SD** | | **CI50±**  **SD** | **CI75±**  **SD** | **CI90±**  **SD** | **DRI50±SD** | | **CI50±**  **SD** | **CI75±**  **SD** | **CI90±**  **SD** | **DRI50±SD** | |
|  |  |  |  | **17AAG** | **CDDP** |  |  |  | **17AGG** | **CDDP** |  |  |  | **17AAG** | **CDDP** |
| **TOV-112D** | **0.52± 0.05** | **0.61± 0.05** | **0.63± 0.07** | 9.74±  1.94 | 3.64± 1.02 | **0.68± 0.06** | **0.67± 0.06** | **0.66± 0.05** | 2.43±  0.30 | 4.50±  0.62 | 1.05±  0.002 | 1.23±  0.01 | 1.24±  0.06 | 97.64±  4.14 | 0.91±  0.07 |
| **TOV-112D**  **Pt-res pool 1** | **0.75±**  **0.05** | **0.76±**  **0.07** | **0.78±**  **0.11** | 3.89± 0.81 | 2.46±  0.86 | **0.73±**  **0.05** | **0.76±**  **0.04** | **0.79±**  **0.02** | 1.77± 0.13 | 4.71±  0.70 | 1.44±  0.10 | 1.76±  0.05 | 2.16±  0.02 | 46.41±  12.98 | 0.61±  0.07 |
| **TOV-112D**  **Pt-res pool 2** | **0.75± 0.07** | **0.72± 0.08** | **0.75± 0.11** | 3.55± 0.51 | 2.37±  0.82 | **0.55±**  **0.04** | **0.61±**  **0.01** | **0.73±**  **0.01** | 2.31± 0.08 | 4.91±  1.59 | 2.16±  0.08 | 1.78±  0.06 | 1.61±  0.13 | 41.84±  5.05 | 0.47±  0.01 |
| **MDAH** | **0.84±**  **0.02** | **0.81±**  **0.04** | **0.82±**  **0.04** | 2.62±  0.29 | 2.01±  0.19 | **0.82±**  **0.04** | **0.82±**  **0.04** | **0.87±**  **0.003** | 1.75±  0.11 | 4.58±  0.44 | 1.04±  0.05 | 1.03±  0.03 | 1.00±  0.03 | 4.29±  0.12 | 1.27±  0.09 |
| **MDAH Pt-res pool 2** | 0.89±  0.04 | 0.87±  0.07 | 0.84±  0.10 | 2.38±  0.57 | 1.69±  0.37 | **0.80±**  **0.02** | **0.84±**  **0.004** | **0.88±**  **0.009** | 1.74±  0.14 | 4.78±  0.16 | 1.14±  0.06 | 1.06±  0.01 | 0.98±  0.02 | 4.29±  0.65 | 1.09±  0.03 |
| **MDAH Pt-res cl. #42** | 0.99±  0.06 | 0.99±  0.04 | 1.06±  0.06 | 2.84±  0.15 | 1.55±  0.18 | 1.05±  0.06 | 1.03±  0.07 | 0.99±  0.07 | 1.54±  0.02 | 2.74±  0.13 | 0.98±  0.07 | 1.14±  0.07 | 1.46±  0.09 | 5.66±  0.09 | 1.12±  0.12 |
| **MDAH**  **Pt-res cl. #12** | **0.81±**  **0.005** | **0.75±**  **0.006** | **0.68±**  **0.04** | 2.54±  0.29 | 2.39±  0.22 | **0.77±**  **0.02** | **0.79±**  **0.002** | **0.79±**  **0.05** | 1.71±  0.02 | 5.29±  1.03 | 1.10±  0.07 | 0.91±  0.02 | 0.83±  0.09 | 4.41±  0.09 | 1.17±  0.05 |

| **STRONG SYNERGISM (CI≤ 0.8 ±SD)** | **SYNERGISM**  **(CI≤ 0.9 ±SD)** | ADDITIVITY  (CI>0.9; ≤1.1 ±SD) | ANTAGONISM  (CI>1.1 ±SD) |
| --- | --- | --- | --- |

CI values (mean±SD) from at least three separate experiments performed in quadruplicate computed at 50% (CI50), 75%(CI75) and 90% (CI90) of cell kill by CalcuSyn software (Biosoft, Cambridge, UK).

CIs values smaller than 0.8 indicate strong synergism highlighted in bold; Cis smaller than 0.9 indicate sinergysm highlighted in bold; additivity (between 0.9 and 1.1) or antagonism (more than 1.1). Different cytotoxic ratios (50:50, 75:25 and 25:75) of each of the two agents were evaluated after 72h.

DRI values (mean±SD) from at least three separate experiments performed in quadruplicate represent the order of magnitude (fold) of dose reduction obtained for IC50 (DRI50) in combination setting compared with each drug alone.

**Supplementary Table 6. Combination index (CI) and dose reduction index (DRI) values for**

**ganetespib and CDDP combination treatment accordingly to different cytotoxic ratio**

|  | **GANETESPIB-CDDP 50:50** | | | | | **GANETESPIB-CDDP 75:25** | | | | | **GANETESPIB-CDDP 25:75** | | | | |
| --- | --- | --- | --- | --- | --- | --- | --- | --- | --- | --- | --- | --- | --- | --- | --- |
| **CELL LINES** | **CI50± SD** | **CI75± SD** | **CI90± SD** | **DRI50±SD** | | **CI50±**  **SD** | **CI75±**  **SD** | **CI90±**  **SD** | **DRI50±SD** | | **CI50±**  **SD** | **CI75±**  **SD** | **CI90±**  **SD** | **DRI50±SD** | |
|  |  |  |  | **GANE** | **CDDP** |  |  |  | **GANE** | **CDDP** |  |  |  | **GANE** | **CDDP** |
| **TOV-112D** | **0.61± 0.06** | **0.67± 0.009** | **0.75± 0.05** | 5.26±  0.19 | 2.24± 0.35 | **0.61± 0.09** | **0.63± 0.03** | **0.62± 0.04** | 2.96±  0.20 | 4.60±  0.25 | **0.50± 0.03** | **0.57±**  **0.01** | **0.66±**  **0.01** | 12.87± 1.19 | 2.45± 0.32 |
| **TOV-112D**  **Pt-res**  **pool 1** | **0.76±**  **0.05** | **0.75±**  **0.07** | **0.82±**  **0.01** | 3.66±  0.06 | 2.25± 0.05 | **0.75± 0.06** | **0.61± 0.009** | **0.48± 0.02** | 2.06±  0.15 | 3.97±  0.21 | **0.61±**  **0.07** | **0.71±**  **0.01** | **0.84±**  **0.04** | 6.84±  0.21 | 2.10± 0.24 |
| **TOV-112D**  **Pt-res**  **pool 2** | **0.80± 0.02** | **0.84± 0.01** | 0.90± 0.01 | 2.71±  0.21 | 2.32± 0.001 | **0.82± 0.03** | **0.60± 0.10** | **0.54± 0.10** | 2.42±  0.08 | 4.99±  0.90 | **0.78±**  **0.004** | **0.78±**  **0.05** | 0.75±  0.19 | 5.38±  0.86 | 1.49± 0.11 |
| **MDAH** | **0.71±**  **0.07** | **0.73±**  **0.07** | **0.76±**  **0.08** | 5.93±  0.70 | 2.10±  0.23 | **0.63±**  **0.09** | **0.67±**  **0.05** | **0.72±**  **0.02** | 2.69±  0.48 | 3.97±  0.43 | **0.78±**  **0.04** | 0.89±  0.06 | 0.91±  0.01 | 11.09±  1.28 | 1.49±  0.16 |
| **MDAH Pt-res**  **pool 2** | **0.77±**  **0.03** | **0.79±**  **0.05** | **0.77±**  **0.06** | 2.47±  0.13 | 2.72±  0.07 | **0.72±**  **0.09** | **0.78±**  **0.04** | **0.79±**  **0.04** | 1.55±  0.17 | 6.41±  0.40 | **0.85±**  **0.04** | 0.86±  0.06 | 0.85±  0.08 | 4.29±  0.43 | 1.52±  0.11 |
| **MDAH Pt-res**  **cl. #42** | 0.97±  0.03 | 0.97±  0.003 | 0.91±  0.06 | 2.51±  0.08 | 1.44±  0.07 | **0.84±**  **0.05** | **0.83±**  **0.02** | **0.79±**  **0.02** | 1.68±  0.17 | 3.56±  0.12 | 1.27±  0.03 | 1.16±  0.09 | 1.22±  0.01 | 5.20±  0.02 | 1.03±  0.20 |
| **MDAH**  **Pt-res**  **cl. #12** | **0.71±**  **0.02** | **0.72±**  **0.02** | **0.74±**  **0.006** | 3.78±  0.19 | 2.25±  0.08 | **0.60±**  **0.0007** | **0.64±**  **0.004** | **0.70±**  **0.01** | 1.96±  0.04 | 4.98±  0.91 | **0.64±**  **0.03** | **0.69±**  **0.01** | **0.79±**  **0.02** | 8.66±  2.01 | 1.66±  0.37 |
| **BJ-hTERT** | 2.29±  0.67 | 1.58±  0.13 | 1.12±  0.14 | 0.79±  0.15 | 1.07±  0.40 |  |  |  |  |  |  |  |  |  |  |

| **STRONG SYNERGISM (CI≤ 0.8 ±SD)** | **SYNERGISM**  **(CI≤ 0.9 ±SD)** | ADDITIVITY  (CI>0.9; ≤1.1 ±SD) | ANTAGONISM  (CI>1.1 ±SD) |
| --- | --- | --- | --- |

CI values (mean±SD) from at least three separate experiments performed in quadruplicate computed at 50% (CI50), 75%(CI75) and 90% (CI90) of cell kill by CalcuSyn software (Biosoft,Cambridge, UK).

CIs values smaller than 0.8 indicate strong synergism highlighted in bold; Cis smaller than 0.9 indicate sinergysm highlighted in bold; additivity (between 0.9 and 1.1) or antagonism (more than 1.1). Different cytotoxic ratios (50:50, 75:25 and 25:75) of each of the two agents were evaluated after 72h except for BJ-hTERT after 96h.

DRI values (mean±SD) from at least three separate experiments performed in quadruplicate represent the order of magnitude (fold) of dose reduction obtained for IC50 (DRI50) in combination setting compared with each drug alone.

**Supplementary Table 7. Combination index (CI) and dose reduction index (DRI) values for**

**ganetespib and doxorubicin combination treatment at equitoxic ratio**

|  | **GANETESPIB-DOXORUBICIN 50:50** | | | | |
| --- | --- | --- | --- | --- | --- |
| **CELL LINES** | **CI50±**  **SD** | **CI75±**  **SD** | **CI90±**  **SD** | **DRI50±SD** | |
|  |  |  |  | **GANETESPIB** | **DOXORUBICIN** |
| **TOV-112D** | **0.51± 0.07** | **0.58± 0.04** | **0.53± 0.02** | 3.69±0.14 | 5.37± 0.62 |
| **TOV-112D**  **Pt-res cl. #2** | **0.66±**  **0.01** | **0.62±**  **0.03** | **0.55±**  **0.04** | 2.32± 0.45 | 4.80±0.40 |
| **TOV-112D**  **Pt-res cl. #7** | **0.69± 0.04** | **0.63± 0.04** | **0.60± 0.04** | 2.45± 0.26 | 3.14±0.45 |

| **STRONG SYNERGISM (CI≤ 0.8 ±SD)** | **SYNERGISM**  **(CI≤ 0.9 ±SD)** | ADDITIVITY  (CI>0.9; ≤1.1 ±SD) | ANTAGONISM  (CI>1.1 ±SD) |
| --- | --- | --- | --- |

CI values (mean±SD) from at least three separate experiments performed in quadruplicate computed at 50% (CI50), 75%(CI75) and 90% (CI90) of cell kill by CalcuSyn software (Biosoft,Cambridge, UK).

CIs values smaller than 0.8 indicate strong synergism highlighted in bold; Cis smaller than 0.9 indicate sinergysm highlighted in bold; additivity (between 0.9 and 1.1) or antagonism (more than 1.1). Equitoxic ratios (50:50) of each of the two agents were evaluated after 72h.

DRI values (mean±SD) from at least three separate experiments performed in quadruplicate represent the order of magnitude (fold) of dose reduction obtained for IC50 (DRI50) in combination setting compared with each drug alone.
